# Topographic maps representing haptic numerosity reveals distinct sensory representations in supramodal networks

**DOI:** 10.1038/s41467-020-20567-5

**Published:** 2021-01-11

**Authors:** Shir Hofstetter, Yuxuan Cai, Ben M. Harvey, Serge O. Dumoulin

**Affiliations:** 1 Spinoza Centre for Neuroimaging, Meibergdreef 75, 1105 BK Amsterdam, The Netherlands; 2 Experimental and Applied Psychology, Vrije University Amsterdam, Van der Boechorststraat 7, 1181 BT Amsterdam, The Netherlands; 3 Experimental Psychology, Helmholtz Institute, Utrecht University, Heidelberglaan 1, 3584 CS Utrecht, The Netherlands

**Keywords:** Perception, Sensory processing

## Abstract

Dedicated maps for cognitive quantities such as timing, size and numerosity support the view that topography is a general principle of brain organization. To date, however, all of these maps were driven by the visual system. Here, we ask whether there are supramodal topographic maps representing cognitive dimensions irrespective of the stimulated sensory modality. We measured haptically and visually driven numerosity-selective neural responses using model-based analyses and ultra-high field (7T) fMRI. We found topographically organized neural populations tuned to haptic numerosity. The responses to visual or haptic numerosity shared a similar cortical network. However, the maps of the two modalities only partially overlap. Thus, although both visual and haptic numerosities are processed in a similar supramodal functional network, the underlying neural populations may be related, but distinct. Therefore, we hypothesize that overlap between modality-specific maps facilitates cross-modal interactions and supramodal representation of cognitive quantities.

## Abstract (web summary)

Topographically organized tuned responses to haptic numerosity were found in the human brain. The responses to visual or haptic numerosity shared a similar large-scale cortical network, yet the maps of the two modalities only partially overlapped, suggesting distinct underlying neural populations.

## Introduction

A core principle of brain organization is a topographic arrangement of neural populations in primary sensory and motor cortices that reflect the structure of their corresponding sensory and motor organs. For example, in the visual cortex, topographic maps preserve the ordered spatial layout of the two-dimensional image that falls on the retina^1,2^. Accordingly, topographic maps are classically considered to be strictly sensory specific. Recently, studies revealed dedicated topographic maps for more abstract cognitive dimensions, which do not depend on the organization of the sensory organs. In particular, these maps showed ordered responses to physical quantities of visual event duration^3,4^, visual object size^5^, and visual numerosity^6,7^, i.e., the set size of items in a group. These findings demonstrate that topography is a common principle of brain organization that goes beyond sensory and motor organ structures. To date, however, all of these cognitive topographic maps were driven by the visual system.

Numerosity perception is an innate ability that guides animal and human behavior^8–14^. Numerical processing relies on tuned, selective neural responses^6,7,15–17^, which may be driven by various sensory modalities^18–20^. The view of a dedicated neural mechanism for number processing that is modality-independent (i.e., “supramodal”) is supported by behavioral^21–26^, developmental^27^, electrophysiological^18^, and imaging studies^28–30^ in animals and humans. Behaviorally, cross-modal numerosity adaptation studies where participants are adapted to stimuli in one sensory modality and tested on another showed perceptual adaptation effects^22,23^. The supramodal representation of numerosity is also reinforced by studies showing no cost-effect for cross-modal numerical judgment^24^, and cross-modal interactions in newborns^27^ and untrained monkeys^21^. Haptic and visual numerosity perception also follow similar response patterns^25,26^.

In line with cross-modal perceptual interactions, neurons in the intraparietal sulcus respond to both auditory and visual numerosity stimuli, in sighted or congenitally blind subjects^28–30^. Electrophysiological recordings in animals have found numerosity-selective responses for auditory and visual stimuli^18–20^. Across the visual and auditory modalities, most neurons showed responses that are sensory-specific, and only a few neurons responded irrespective of sensory modality^18^.

Here, we ask whether topographic maps of supramodal cognitive networks either depend on the driving sensory modality or represent features irrespective of the driving sensory modality. To that end, we utilize the recently uncovered network of visual topographic numerosity maps^6,7^ and extended our search to the haptic domain. First, we test whether numerosity topographic maps can also be driven by other sensory modalities, specifically by the haptic system. We hypothesize that the principle of minimizing neural wiring length between neurons with similar tuning properties will result in topographic maps also for neurons tuned to haptic numerosity^31,32^. Then, we test whether the numerosity selectivity of the neural populations within these maps is modality independent. To that end, we compared within-participant their haptic and visual topographic maps.

## Results

### Neural populations are tuned to haptic numerosity

**Fig. 1:**
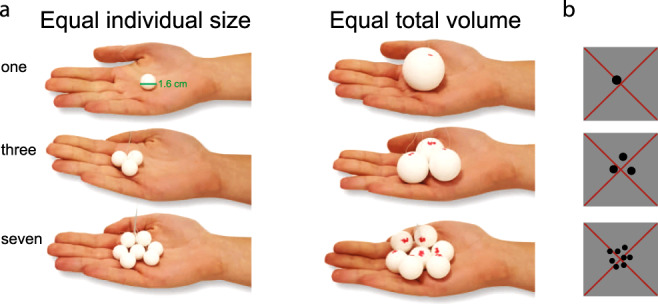
Illustration of the stimuli. **a** Static images show the two different haptic stimulus conditions, with examples of different numerosities. In the equal individual size condition, each sphere was the same size irrespective of the number of spheres. So, the total volume of the spheres increased with increasing numerosity. In the equal total volume condition, the total volume of all spheres was kept constant. So, as numerosity increased the individual sphere size decreased. **b** Examples of the visual stimulus at different numerosities. The total surface area of the dot pattern remained the same across numerosities. A large, thin, red fixation cross passes diagonally through the center of the display, and through the center of the dot pattern.

We placed different numbers of spheres (1–7, with a baseline of 20 spheres) in the right hands of participants while collecting ultra-high field (7T) functional magnetic resonance imaging (fMRI) data (Fig. 1a). We tested two stimuli conditions in order to control unavoidable relationships between numerosity and either individual object size or total object volume (or weight)^33–35^. The equal individual sphere size condition included spheres of the same size, irrespective of the number of spheres. In this condition, the total volume of the spheres increased with increasing numerosity. The second condition of equal total volume kept the total volume of spheres constant. Here, as numerosity increased the individual sphere size decreased. The spheres were put in the participant’s hand for 3 s and were replaced during an interstimulus break of 4.5 s. The difficulty of numerosity judgments increases with numerosity and may therefore confound our results. Thus, the participants were not instructed to judge the presented numerosity but were asked to explore the spheres while they were in their hands.

**Fig. 2:**
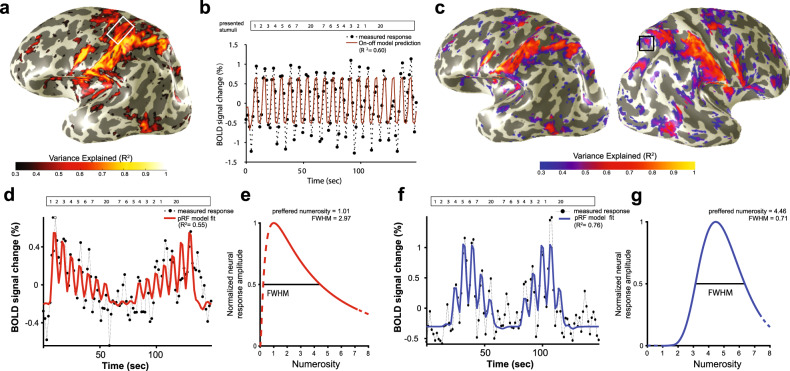
Neural responses to haptic numerosity exploration. **a** Responses elicited by the haptic exploration of the numerosity stimuli and captured by the numerosity pRF model (variance explained). These include responses in the left motor cortex (inset). The variance explained by the numerosity model (*R*^2^) is derived across the two stimulus conditions (see Fig. 1a). **b** Example of the fMRI time-series from the left motor cortex illustrates similar responses throughout the task, irrespective of numerosity. The numerosities are indicated at the top of the graph. Due to the relatively slow stimulus presentation, the fMRI time-series reveals responses to each individual stimulus presentation. This pattern of responses can be well captured by a general linear model where the presence of the spheres serves as a predictor (solid line). **c** Goodness of fit of numerosity-selective neural models after removal of cortical locations where the general motor predictions do not differ from numerosity selective response predictions (see methods). **d**, **f** Two samples of fMRI time courses from sites in the right posterior parietal cortex (black box in **c**). The two sites show distinct responses to different haptic numerosities: **d** the largest response amplitude occurs at low numerosities; **f** the largest response occurs at higher numerosities. The numerosity pRF model’s predictions (solid lines) capture these different responses as different numerosity selectivity parameters. **e**, **g** The pRF model summarizes the neural responses using a logarithmic Gaussian tuning function with two parameters: preferred numerosity and tuning width, defined by the full width at half maximum (FWHM). Both cortical locations respond strongly to the stimulus manipulation but with different preferred numerosities.

We fitted a population receptive field (pRF) model of numerosity selective responses with two parameters: preferred numerosity and tuning width^6,7^ (Fig. 2a). We distinguished between neural responses selective to numerosity and to the “on-off” presence of stimuli in the hand and the subsequent exploration, irrespective of its numerosity (see “Methods”; Fig. 2b). The pRF model explained a large amount of the signal variance (>50%) in similar areas to those we previously reported for visual numerosity responses^7^: the occipital–parietal, occipital–temporal, parietal, and posterior–superior frontal lobe (Fig. 2c, Supplementary Fig. 1). Within these regions, the pRF model reveals neural populations tuned to haptic numerosity (Fig. 2d–g). The consistent activation of these regions in our numerosity studies suggests they are part of a network. We assume they are connected, or at least connected to the same origin, resulting in related activation during our numerosity presentations.

### Tuned haptic numerosity responses are organized in a network of topographic maps

**Fig. 3:**
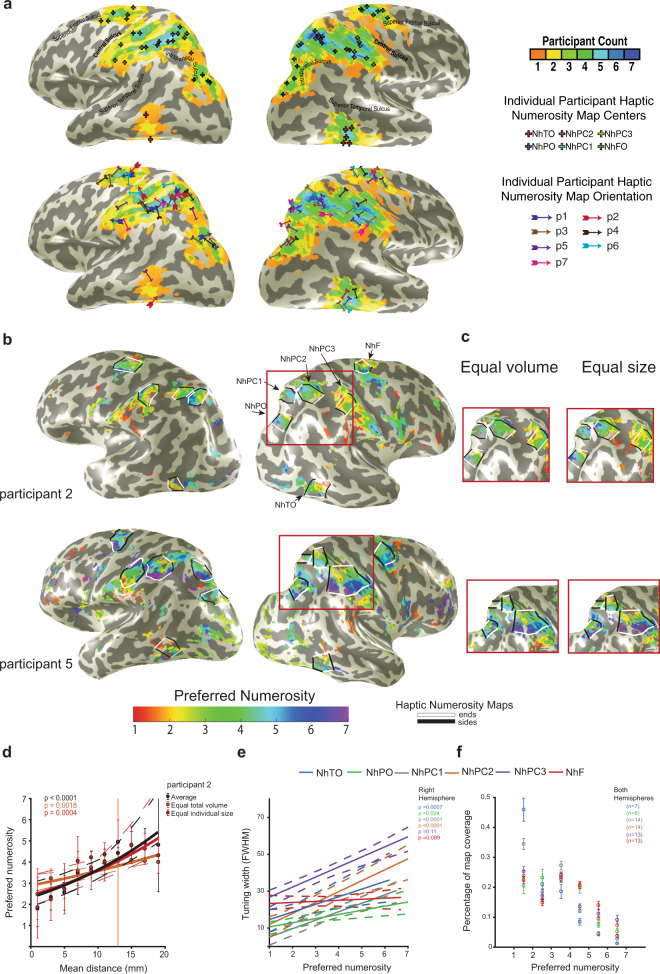
Haptic-driven topographic numerosity maps. **a** Haptic numerosity map locations of all participants transformed onto the N27 (Talairach) template’s cortical surface anatomy. Colors indicate the overlap between the maps of all participants. Crosses show the transformed locations of individual participants’ map centers (top). Arrows indicate the gradient’s directions of each map (bottom). **b** Numerosity preferences for data averaged from both haptic stimulus conditions of two example participants (variance explained > 30%). Colors represent preferred numerosity. Although only the right hand was used, several bilateral topographic maps were found consistently across participants. The borders of lowest to highest preferred numerosity in each map are marked by white lines. Black lines complete the margins of the maps. **c** Maps in the parietal area are shown from two example participants for each haptic stimulus condition of equal total volume and equal individual sphere size. **d** Preferred numerosities within NhPC1 map of participant two plotted as a function of the distance along cortical surfaces (measured between the white lines, see panel **b**). The preferred numerosity increases systematically and repeatably in the two stimuli configurations. Solid lines show logarithmic fits with their 95% confidence intervals (dashed lines) determined by bootstrapping (10,000 iterations). The colored text gives the probability of the observed change from permutation analysis. Error bars show the standard error of the mean for each data point. Number of recording sites across the data points: *n*(min) = 4, *n*(max) = 17, *n*(mean) = 11.8. **e** Increase in tuning widths plotted as a function of the preferred numerosity, averaged across participants. Solid lines show linear fits with their 95% confidence intervals (dashed lines) determined by bootstrapping. The text gives the probability of the observed change from permutation analysis for each map. In 4/6 maps, tuning width increased significantly with preferred numerosity. **f** Percentage coverage of the map plotted as a function of preferred numerosity. Grouped across participants and hemispheres, we found a decrease in cortical magnification at higher numerosities, i.e., more cortical area prefers lower numerosities. Error bars show the standard error of the mean. *n* indicates the number of maps that were grouped together.

Overall, six topographic haptic-numerosity maps were found in each hemisphere (Fig. 3a, b). The averaged variance explained in these maps was 0.53 (corresponding to *p* = 0.00087 uncorrected, see “Methods”). In each map, the numerosity preference progressed systematically along the cortical surface, repeatedly across participants and stimulus configuration (Fig. 3b, c, Supplementary Figs. 2–4). To distinguish these maps from the visual numerosity network previously reported^7^, we added “h” for haptic. The most posterior map, NhTO, lay at the inferior temporal gyrus. At the superior end of the parieto-occipital sulcus, lay the second map, NhPO. Three maps (NhPC1, NhPC2, and NhPC3) lay around the postcentral sulcus. NhPC2 lay superiorly to NhPC3 at the postcentral sulcus. The sixth map (NhF) lay at the frontal cortex at the posterior end of the superior frontal sulcus. For the center position of the maps in Montreal Neurological Institute (MNI) coordinates^36^ please see Supplementary Table 1.

Based on the average over both stimulus conditions, 37/42 (88%) maps were defined in the right hemisphere, and 32/42 (75%) maps were defined in the left hemisphere. The change in numerosity preference in each map was quantified by measuring the distance of each data point from the borders of the map with the lowest and highest preferred numerosities (white lines, Fig. 3b and Supplementary Fig. 2). Preferred numerosity was plotted against cortical distance (Fig. 3d and Supplementary Figs. 5 and 6). On average over both conditions, 36/37 (97%) left hemisphere and 31/32 (97%) right hemisphere maps showed a significant progression of preferred numerosity across the cortical surface. In the individual conditions (i.e., equal total volume and equal individual sphere size) 69/74 (95%) left hemisphere and 60/64 (94%) right hemisphere maps showed a significant progression (Supplementary Table 2).

Preferred numerosity within each map was well correlated between stimulus configurations of the equal total volume of spheres and equal individual sphere size (Supplementary Figs. 7 and 8). This correlation between stimulus conditions was significant in 79% of the maps (28/37 and 26/32 maps in the right and left hemisphere respectively, false-discovery rate (FDR)-corrected for multiple comparisons), demonstrating repeatable map organization across stimulus conditions.

Next, we evaluated two known organizational properties of topographic sensory maps. First, we estimated whether numerosity tuning width in the responses averaged across haptic stimulus conditions changed significantly with preferred numerosity. Averaged across participants, numerosity tuning width increased significantly with preferred numerosity in 4/6 right hemisphere maps, and 5/6 left hemisphere maps (Fig. 3e and Supplementary Fig. 9).

Another known feature of sensory maps relates to the proportion of their cortical surface dedicated to processing specific areas of sensory organs, i.e., the cortical magnification factor. We find a cortical magnification effect where more cortical surface sites prefer lower than higher numerosities (Fig. 3f). Grouped across participants and hemispheres, all the maps exhibit a significant decrease in cortical surface with preferred numerosity (NhTO: *p* = 0.000001, *t*(36) = 5.58; NhPO: *p* = 0.001, *t*(42) = 2.2; NhPC1: *p* = 0.0000001, *t*(78) = 5.56; NhPC2: *p* = 0.006, *t*(78) = 2.54; NhPC3: *p* = 0.01 *t*(72) = 2.32; NhF: *p* = 0.005, *t*(72) = 2.61, FDR corrected for multiple comparisons). These two factors of increased tuning width and decreased cortical surface devoted to higher numerosities were also found in the visual numerosity maps^6,7^ and may explain the decreased behavioral precision of higher numerosities^6,17^. We propose that similarities between haptic and visual numerosity perception may be partially reflected by similar properties of the haptic and visual numerosity maps. Furthermore, estimation of haptic and visual numerosity may rely on related processes.

### Fingers motion does not vary during haptic exploration of numerosity

We next set to explore whether our haptic exploration task requires increased finger motion with increase numerosity, which may confound our results of tuned haptic neural responses. Therefore, we tested five participants who laid in a mock scanner with their eyes closed, wearing on their right hand a glove that captures fingers motion (5DT Glove Ultra, Fifth Dimension Technologies). Three of these participants also participated in the haptic experiment in the scanner. We repeated exactly the same experiments: spheres ranging from one to seven (with a baseline of twenty spheres) were placed in the participant’s hand for 3 s by an experimenter. The spheres changed every 4.5 s. Identical to the fMRI experiments, the participants were asked to explore the spheres, no numerosity judgment was required. We evaluated both haptic stimulus conditions (equal total volume and equal individual sphere size). Though the glove reduces tactile sensitivity, as texture information is missing, the active nature of the task (i.e., exploring the spheres while they are placed at the hand) should be comparable to the original task performed in the scanner, allowing us to evaluate whether fingers motion changes with numerosity.

**Fig. 4:**
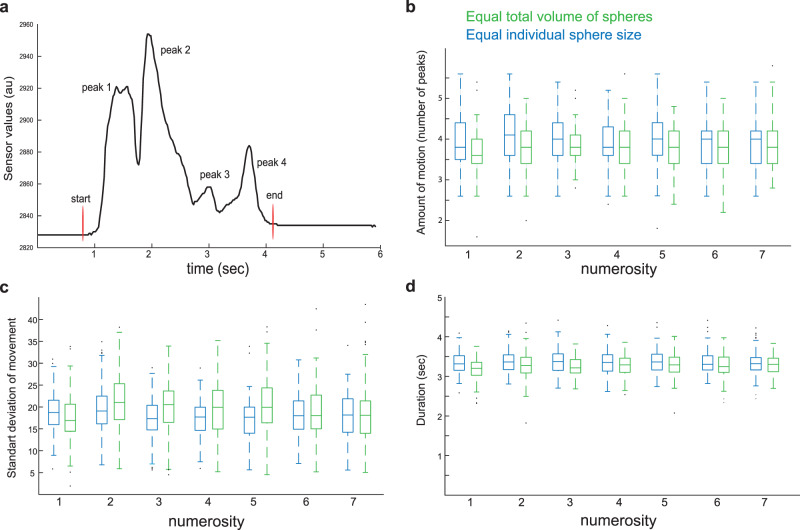
Motor properties of the exploration task did not vary with haptic numerosity. **a** Hand motion during haptic exploration of spheres was recorded using a glove that captures fingers motion. The epochs of spheres exploration were extracted for each finger. For each epoch, we measured **b** the number of unique motions as indicated by the number of peaks in the sensor values **c** the total amount of motion as indicated by the standard deviation of sensor values, and **d** the duration of motion as indicated by the start and end of the changes in sensor values irrespective of the beginning and end positions. Box plot diagrams present these parameters averaged across fingers and grouped across haptic epochs of all participants (*n* = 100). We did not find any difference in these parameters as numerosity increased, in either of the two haptic stimuli conditions of equal total volume or equal individual sphere size. Indeed, Bayesian statistics provided evidence that there are no differences in these parameters as a function of numerosity. In the box-plot diagram, the central mark indicates the median, and the bottom and top edges of the box indicate the 25th and 75th percentiles, respectively. The whiskers extend to the most extreme data points not considered outliers, and the outliers are plotted individually using the dot symbol.

We quantified the amount of finger motion in each sphere exploration epoch (Fig. 4a) by three parameters: the number of unique motions, total variation of motion, and duration of motion. The amount of motion was calculated by the number of distinct movements as indicated by the number of peaks, the total variation of motion was the standard deviation (STD) of the signal, and the duration of motion as indicated by the start and end of the changes in sensor values irrespective of the beginning and end positions (Fig. 4a).

There were no significant differences between the number of unique motions, total amount of motion, or the duration of motion with numerosity (two-way repeated measured ANOVA—haptic stimulus conditions × numerosity; the difference in the number of unique motions: *F*_(6,24)_ = 0.379, *p* = 0.885; differences in variation of motion: *F*_(6,24)_ = 0.389, *p* = 0.879; on motion duration: *F*_(6,24)_ = 2.479, *p* = 0.052; motion duration: *F*_(6,24)_ = 2.479, *p* = 0.052). Furthermore, Bayesian repeated-measures ANOVA indicated evidence for the null hypothesis, i.e., that the number of unique motions, the total amount of motion, and motion duration do not change with numerosity (BF_01_ = 3581.1, BF_01_ = 15.6, and BF_01_ = 168,728 for analyses of the amount of motion, its variation and duration, respectively. Prior probabilities of each model P(M) = 0.2). We also failed to find a significant difference in the average amount of motion (*F*_(1,4)_ = 6.4, *p* = 0.064) and its variation (*F*_(1,4)_ = 1.8, *p* = 0.246) under the two haptic stimulus conditions. In summary, the participants moved their right hands to explore the spheres in a similar fashion for all numerosities, and consequently, we suggest that differences in hand motion cannot explain the tuned responses for numerosity in the current haptic experiment.

### Haptic and visual-driven neural responses share a similar cortical network

**Fig. 5:**
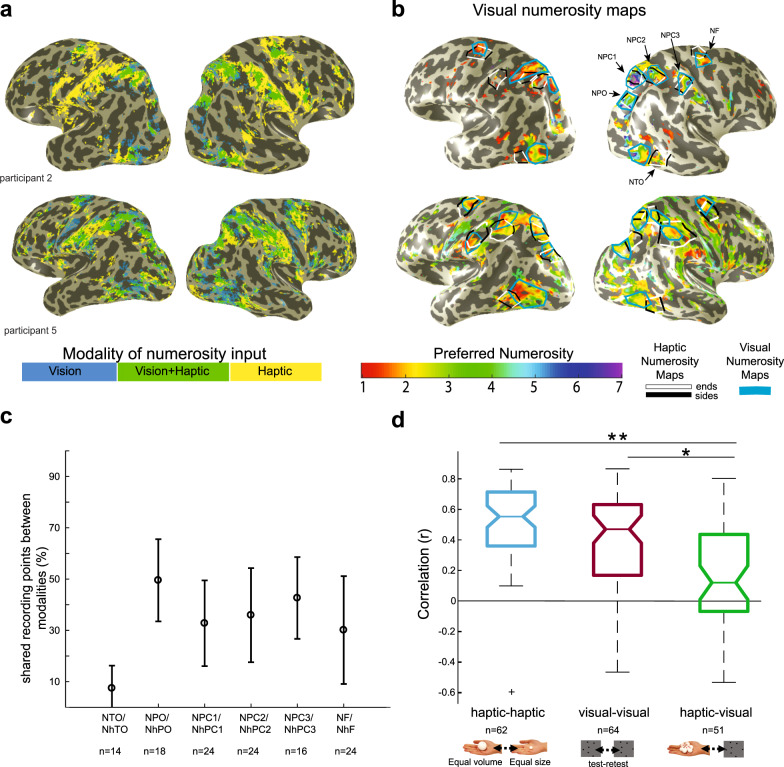
Numerosity-selective responses are modality-specific. **a** Neural responses driven by visual and haptic numerosity stimuli largely overlap. Colors in each data point represent which type of sensory input produced numerosity-selective neural response (as captured by the pRF model with variance explained >30%). **b** Maps of preferred visual numerosities with the outlines of the visual and haptic numerosity maps shown in blue and black/white lines respectively. The topographic numerosity maps of the two modalities only partially overlap. **c** Percentage of overlap between the visual and haptic numerosity maps relative to the map sizes averaged across participants, hemispheres, and modalities (i.e., visual and haptic numerosity maps). Error bars show the standard deviation of the mean. *n* indicates the number of maps that were averaged together. **d** No systematic relationship between the two modalities was found between participants or maps. A box-plot diagram shows the distribution of correlation coefficient values (*r*) that were obtained from the analyses of correlation between the two haptic stimulus configurations, the visual test–retest and the correlation between preferred haptic and visual numerosities in their shared areas (right). Stars show significant differences between groups in post hoc test for multiple comparisons (Bonferroni correction; * indicates *p*_adj_ = 2 × 10^−^^5^; ** indicates *p*_adj_ = 1.9 × 10^−9^). The average of the correlation coefficients of each part of the data was significantly higher than zero (one-sample *t* test: two haptic stimulus configurations: *t*(61) = 14, *p* = 9.8 × 10^−^^21^; visual test–retest: *t*(63) = 9.75, *p* = 3.2 × 10^−14^; haptic and visual: *t*(50) = 3.18, *p* = 0.0025). In the box-plot diagram, the central mark indicates the median, and the bottom and top edges of the box indicate the 25th and 75th percentiles, respectively. The whiskers extend to the most extreme data points not considered outliers, and the outliers are plotted individually using the “+” symbol. *n* indicates the number of maps that were grouped together.

Next, we compared the network of haptic numerosity maps with the visually driven numerosity maps. We projected onto the reconstructed hemispheres sites which exhibit numerosity selectivity (with variance explained over 30%) for visual, haptic, or both modalities. In general, shared selectivity for visual and haptic numerosity was found in a similar network of regions in the parietal and frontal cortices (Fig. 5a and Supplementary Fig. 10). Responses to haptic numerosity alone were found in larger parts of the postcentral sulcus.

### Distinct haptic and visual-driven numerosity topographic maps

We then overlaid the borders of the haptic numerosity maps on top of the maps of the visual numerosity preferences. This overlay revealed that the haptic and visual numerosity maps only partially overlap (Fig. 5b and Supplementary Fig. 11). The overlapped area of shared cortical points between the maps relative to the sizes of each map was 34% with an STD of 23.9% (Fig. 5c and Supplementary Fig. 12). The shared area between the numerosity maps in the temporal-occipital area (i.e., NTO/NhTO) was significantly smaller than the shared areas between the visual and haptic maps in the parietal and frontal cortices (one way ANOVA; *p* = 0.0000051, *F*(5,114) = 8.08 followed by post hoc analysis, Bonferroni correction for multiple comparisons).

Only in regions where the maps of the two modalities overlap, we calculated the correlation between the numerosity preferences of the haptic and visual stimuli (For each map and each participant, see Supplementary Figs. 13 and 14). Therefore, this analysis is biased towards showing a relationship between modalities, as it does not take into account the neural preferences of numerosity in the areas that are not shared between the maps. This analysis showed a large variability between maps and participants. We compared the distributions of the correlation coefficients obtained from the between modalities analysis and the within modalities analyses (i.e., between maps of haptic stimulus configurations of the equal total volume of spheres and equal individual sphere size, and between visual maps test–retest). The means of the distributions were significantly different (one-way ANOVA: *p* = 2.7 × 10^−9^, *F*_(2,174)_ = 22.12). The mean of the correlation coefficients between modalities was significantly lower than the means of distributions of correlation coefficients within modalities (post hoc analysis, Bonferroni correction for multiple comparisons; haptic-vision correlations contrasted with vision test-retest: *p*_adj_ = 0.0002; haptic-vision correlations contrasted with the correlation between the two haptic conditions: *p*_adj_ = 1.9 × 10^−9^).

Some correlation is expected when two independent gradual progressions are overlaid unless their direction of progression is orthogonal. However, the higher variability and lower average of correlation demonstrate less relationship between the progressions of the points shared between the maps of the two modalities. Overall, the two parameters of the low percentage of cortical locations responding to both modalities and the low correlation of numerosity preferences among these locations suggest that the topographic visual and haptic numerosity maps are distinct. However, the general overlap between the visual and haptic numerosity networks, as well as the significant (although lower) correlation between numerosity preferences between the two modalities suggest that the two networks are related, and some neural populations may be connected.

### Similar behavior in visual and haptic numerosity perception

Lastly, we tested whether differences in stimulus design between the haptic and visual numerosity experiments may explain the apparent dissociation between the topographic maps of the two modalities. It is well-known that the perception of small numerosities, usually up to four, is rapid and accurate. As numerosity increases, accurate estimation of numerosity is longer and more error-prone. This behavioral dissociation originally proposed two separate processes of numerosity representation: the “subitizing” range for numerosity up to four and the “counting” range for larger numerosities^37–41^. Here, we asked whether the duration of stimuli presentation (300 ms in the visual experiment and 3 s in the haptic experiments) allowed for different mechanisms of numerosity perception between the two sensory modalities.

To address this question, we tested numerosity perception in the haptic and visual domains. In the haptic experiment, we placed different numbers of spheres (one to seven, using both stimulus conditions) in the right hands of five participants (one of the participants was also included in the fMRI experiments). The participants were asked to judge the number of spheres they explored. The spheres were set for 3 s in a randomized order.

**Fig. 6:**
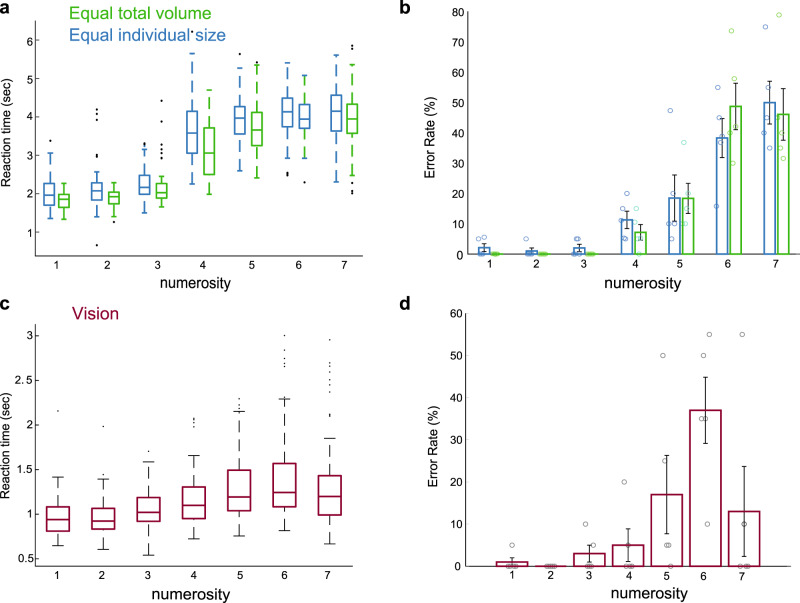
Numerosity judgment with increased numerosity. **a** Participants were asked to judge the number of spheres placed in their hands for three seconds. A box-plot diagram shows the distribution of reaction times of all participants for each numerosity (*n* = 100 for each numerosity). A similar pattern of increase in reaction time is found for each stimulus condition of the equal total volume of spheres and equal size of individual spheres. **b** Bar plot presents the percentage of errors in judgment of numerosity averaged across participants (represented by individual dots) and for each haptic stimulus condition. Error bars show the standard error of the mean. Numerosities above the subitizing range are more prone to error. **c** A box-plot diagram shows the distribution of reaction times of all participants for numerosities that were visually displayed for 300 ms (*n* = 100 for each numerosity). A significant increase in reaction time was found with increased numerosity. **d** Bar plot shows the percentage of error in judging visual numerosity, averaged across participants (represented by individual dots). Error bars show the standard error of the mean. Above the subitizing range, numerosity judgment is less accurate. Overall, the perception of numerosities in the haptic and visual domains show similar behavior in terms of reaction times and error rates. In the box-plot diagram, the central mark indicates the median, and the bottom and top edges of the box indicate the 25th and 75th percentiles, respectively. The whiskers extend to the most extreme data points not considered outliers, and the outliers are plotted individually using the dot symbol.

Reaction time significantly differed (increased) with numerosity in both haptic stimuli conditions (Friedman’s test: equal individual spheres size: *p* < 0.0001, *χ*^2^(6) = 27.6; equal total volume: *p* < 8.9 × 10^−5^ *χ*^2^(6) = 28.11; Fig. 6a). Error rate also differed with numerosity. The error rate for numerosities one to three were significantly lower than error rates for numerosities ranging from five to seven, in both haptic stimulus conditions (chi-square tests between pairs of numerosities, followed by Bonferroni correction for multiple comparisons, *p*_adj_ < 0.002; Fig. 6b).

Our results are in agreement with a similar haptic exploration study by Plaisier et al.^25^, and shows that haptic exploration follows a known pattern of the immediate and error-free judgment of numerosities one to three (i.e., the “subitizing range”). As shown in Fig. 6b, estimation is less accurate for numerosities above three. Importantly, 3 s of haptic exploration is not enough to accurately judge the numerosity of more than four spheres.

A similar pattern of increased reaction time and error-rate with numerosity was found in a visual experiment. Here, the same participants were asked to judge the amount of randomly grouped dots displayed on a screen for 300 ms (Fig. 6c, d; Fig. 1b presents stimuli examples). Reaction time differed (increased) with numerosity (Friedman’s test *p* = 0.0002, *χ*^2^(6) = 25.89). Error rates for numerosities one to three significantly differed from numerosities five to six. (Chi-square tests between pairs of numerosities, followed by Bonferroni correction for multiple comparisons, *p*_adj_ < 0.05). Accuracy, however, improved for numerosity of seven. This improvement might reflect the boundary effect of the range being tested (i.e., seven was the maximum tested numerosity). Therefore, participants might have been more likely to link the maximum stimulus presented with the maximum tested numerosity, because they could not overestimate this numerosity.

Finally, we also tested whether the pattern of change in reaction time and increase in error rate with numerosity was similar between the visual and haptic experiments. Both reaction times and error rate were well correlated between the visual and haptic domains (Spearman correlation for reaction times: vision and equal total volume of spheres: *r*_s_ = 0.57 *p* = 0.00042; vision and equal individual sphere size: *r*_s_ = 0.61 *p* = 0.00011; Spearman correlation for error rates: vision and equal total volume of spheres: *r*_s_ = 0.58 *p* = 0.00022; vision and equal individual sphere size: *r*_s_ = 0.49 *p* = 0.0028).

Overall, these results suggest that in the current study design numerosity perception was similar between the two sensory modalities. Specifically, in both visual and haptic experiments the stimuli were not presented long enough for accurate counting of numerosities above the subitizing range. Thus, the discrepancy between the visual and haptic topographic maps could not be attributed to differences in stimuli presentation times.

## Discussion

We found a network of neural populations tuned to haptic numerosity of items explored in the hand. These are topographically organized by their preferred numerosity. They exhibit common features of sensory processing, such as systematic changes in tuning width with preferred numerosity and a larger extent of cortical surface preferring lower numerosities (i.e., cortical magnification). At the macroscale, largely overlapping occipital, parietal and frontal neural populations responded to both visual and haptic numerosity. However, at the finer scale, the visual and haptic numerosity maps were distinct. Thus, although numerosity is processed in a supramodal functional network, the responses of the underlying neural populations are primarily modality-specific. Nevertheless, many studies show cross-modal interactions in numerosity perception^22–24^. Our results also indicate some relationship between the neural populations tuned to each modality. Therefore, we speculate that the overlap between the modality-specific numerosity-tuned neural populations may allow generalization and interactions between distinct numerosity representations, without mapping these onto a common metric.

Many studies describe neural responses to visual numerosity^10,12,15–18,29,30,42^. Others describe responses to temporal numerosity (the number of events, rather than the number of items) in vision, audition, or motor sequences^43,44^. Here we show selective neural responses to the number of items haptically explored. In the tactile domain, few behavioral studies examine parallels between tactile or haptic numerosity perception and visual numerosity perception. A tactile or haptic subitizing range, where numerosity judgment is immediate and accurate, has been described for both passive perceptions of vibrotactile sensation to the fingers^45^ and active exploration of spheres placed in the hand^25^. We have also found immediate and accurate judgment of haptic exploration for numerosities in the subitizing range (Fig. 6a, b). Moreover, we show that even though the stimulus presentation time was relatively long in comparison to the visual domain, a similar increase in reaction time and the error rate is found when judging numerosities above the subitizing range. The haptic numerosity-selective populations we describe may provide a neural mechanism for such haptic numerosity perception: neural responses to visual numerosity predict numerosity perception in macaques^16^, children^46^, and adults^47,48^.

As haptic exploration is an active process, haptic numerosity selectivity may reflect somatosensory and/or motor components of the task, consistent with postcentral and precentral responses, respectively. However, the numerosity tuned responses were distinct from contralateral primary somatosensory and motor responses (Fig. 2a, b). In addition, a similar amount, variation, and duration of fingers motion were recorded during the haptic exploration task for all numerosities (Fig. 4). Therefore, increased motion with numerosity could not explain our haptic tuned response. Moreover, though the spheres were placed only at the right hand (the dominant hand for 6 of the 7 participants) similar haptic numerosity-selective responses were found in both hemispheres. Finally, the duration of the presentation was sufficient for exploration of the entire set (but not for counting) and did not require any quick or skilled motor responses specific to the dominant hand. Thus, the haptic numerosity tuned responses we observe here could not be explained by low-level motor properties, laterality of stimulus presentation, or the use of a dominant hand. Notably, we do not attempt to distinguish the sensory and motor properties of haptic numerosity perception. Both sensory and motor aspects, and their interaction, are plausible candidates to drive the tuned neural responses we found. The nature of this mechanism is still unknown. It may be that higher-order processes are involved in the motor and sensory exploration strategies that ultimately give information about the haptically “sensed” numerosity. Further research is needed in order to disentangle the relationship between the sensory and motor components underlying haptic numerosity perception, and the mechanisms by which haptic numerosity tuned responses are computed.

Some differences exist in our experimental design between the visual and haptic numerosity presentations, as it is not possible to use identical stimuli and tasks in the two sensory modalities. Therefore, as was done in the first paper to study the topographic representation of visual numerosity^6^, we tried to account for the effect of differences in stimuli properties that may covary with numerosity within a modality. We found a good agreement in the progression of the haptic numerosity maps between the two haptic stimulus conditions of constant sphere size and constant total volume of spheres. Between modalities, we compared responses under similar stimulus properties: Both stimulus sets included stimuli where individual object size was constant across numerosities (for visual domain this is presented in ref. ^6^) and where total object area (for visual) or total object volume (for haptic) was constant. The density of the stimuli also differed somewhat between modalities: visual displays had evenly spaced patterns with some space between objects, while haptic groups were placed into the hand in a bunch where the spheres touched. However, we have previously demonstrated that density does not affect neural numerosity responses in visual displays^6^. The density of the haptic group (i.e., spatial extent divided by numerosity) changed in different ways with numerosity between the two haptic stimulus configurations and produced very similar responses. So the responses to numerosity do not seem to follow density, and density differences within a modality do not seem to affect numerosity selectivity. Therefore, density differences between modalities are unlikely to explain changes in numerosity tuning. With regard to differences in numerosity perception between modalities, we have found a similar pattern of increased reaction time and error rate for numerosities above the subitizing range, which also correlated across modalities. Finally, the attentional set could not be matched across modalities, largely because participants must attend to the input from different sensory systems. The attention focused on a stimulus is an inherent part of the neural responses to that stimulus. Nonetheless, differences in attention alone are unlikely to drive the changes in map orientations that were found between modalities. We, therefore, suggest that the distinct visual and haptic topographic maps present neural responses to numerosity captured by each sensory modality and that the distinction between the maps could not be explained by differences in perceptual properties, attention, or stimulus set.

The extent to which the perception and neural representation of numerosity are modality-independent has been highly debated over the years^49^. Evidence for a supramodal numerosity representation comes from cross-modal adaptation studies which found numerosity adaptation effects when the adapted numerosity was presented in one modality (e.g., vision) and the test numerosity in another (e.g., audition)^22^. A similar cross-modal adaptation effect was found between fingers tapping and visual numerosity perception^23^. Infants also preferentially attend to stimuli matched for auditory, visual^27,50^, and tactile numerosity^51^. Such perceptual interactions are generally assumed to rely on transforming numerosity information from different modalities into a common, supramodal neural representation. However, it is equally possible that modality-specific numerosity representations interact. Other studies find that the accuracy of numerosity perception differs between modalities^52^. Macaque electrophysiological evidence advocating a supra-modal representation of temporal numerosity^18^ finds only 3% (6 neurons) of recorded parietal neurons and 11% (25 neurons) of recorded frontal neurons responded to both auditory and visual input^18^. On one hand, our results agree with and extend these electrophysiological findings by showing that most spatial numerosity-selective responses are modality dependent. On the other hand, our results suggest that the modality dependent neurons are found in close vicinity of each other in overlapping topographic maps. We, therefore, propose that perceptual interactions may reflect interactions between these nearby distinct neural populations. Notably, the maps in the occipitotemporal area (NTO/NhTO) show significantly less overlap between modalities than the maps in the parietal and frontal cortices. We speculate that the interaction between modality-dependent populations may be mostly formed in the parietal and frontal association areas.

Another hypothesis, which is not mutually exclusive, suggests that a supramodal representation of numerosity results from subsequent convergence of information from the different modalities. Conceptually, this is in line with macaque electrophysiological results^53^ showing two distinct neural populations tuned to spatial and temporal visual numerosity, with the proportion of neurons responding to the same numerosity in both protocols being at chance level. After enumeration was complete and the animal was preparing to compare either protocol to a spatial numerosity display, a third population began exhibiting protocol-independent numerosity selectivity. This suggests that multiple processing steps may be needed to give a supramodal numerosity representation. But it is also possible that these supramodal (visual and auditory) and format-independent (spatial and temporal) numerosity-selective responses rely on training to compare all of these stimuli to the same visual-spatial numerosity display. We may not see such generalization as our participants made no numerosity judgments or comparisons between modalities, and are not trained to generalize. But we may also miss a potential supramodal subpopulation of neurons because fMRI averages neural responses across the neural populations within each voxel, so we cannot differentiate the contributions of individual neurons. If such a population exists, it may be represented by its own network of topographic maps which should be in close proximity to the two networks we already uncovered. Alternatively, supramodal numerosity representations may be coded in patterns of activations, something that may be revealed by MVPA analyses or multiunit neurophysiology.

The network of haptic numerosity maps provides further evidence that topography is a common principle of brain organization, allowing minimization of neural wiring^31^. The representation of cognitive dimensions, such as numerosity, is not restricted to the visual modality alone. Although the organization of the numerosity maps does not follow the organization of a sensory organ, neural responses within these maps are primarily specific to a sensory modality. Nevertheless, overlap and interactions between modality-specific maps may facilitate cross-modal interactions and subsequent generalization.

## Methods

### Participants

Eleven individuals participated in the study. Seven individuals participated in the haptic numerosity study conducted in the MRI (1 female, 1 left-handed, mean age 33, age range: 26–46). Out of these seven participants, six participants were also scanned in the visual numerosity experiment. Five individuals participated in a movement control experiment (2 males, 1 left-handed, mean age 32, age range: 25–38). Behavioral tests of haptic and visual numerosity perception included five participants (1 male, 2 left-handed, mean age 29, age range: 25–35). All participants had normal or corrected to normal visual acuity and normal haptic perception. All participants gave informed consent. All experimental procedures were cleared by the ethics committee of University Medical Centre Utrecht.

### Haptic numerosity stimuli

We placed varying numbers of plastic spheres (1–7, with a baseline of 20 spheres, Fig. 1a) in the right hands of participants. The spheres were all either the same size (equal size condition, where each individual sphere was 21 mm^3^) or the same overall volume (equal volume condition, where the total sum of all spheres was 420 mm^3^). The spheres were suspended from plastic wires.

The haptic numerosity experiment included exploration periods of 3 seconds. Another set was placed in the hand after 4.5 s, i.e., the inter-stimulus interval. Participants laid in the scanner with their eyes closed and were asked to explore the spheres, but no numerosity judgments were required. The spheres were placed in the hands of the participant by an experimenter in the scanning room. The experimenter received auditory cues (through headphones the participant could not hear) indicating which numerosities to present at each point and when to place or remove the spheres from the hands of the participants. Numerosities 1–7 were presented in ascending order, followed by three presentations of 20 spheres. Then, numerosities 7 to 1 in were presented in descending order, followed by a similar three-time presentation of 20 spheres. This pattern was repeated 2 times during a functional run.

### Hand motion control

In order to exclude fingers motion as a confound of haptic numerosity neural preferences, we conducted a similar haptic numerosity experiment using a glove that captures hand motion at around 52 Hz (5DT Glove Ultra, Fifth Dimension Technologies). The glove measures fingers flexure and the abduction between the fingers. In this experiment, five participants laid in a mock scanner with their eyes closed, wearing the glove on their right hand (three participants out of the five also participated in the haptic experiment in the MRI scanner). Mimicking the experiment that was performed in the MRI, an experimenter placed spheres in the right hands of the participants for 3 s, with a 4.5 s interstimulus interval. The spheres were presented in the same ascending and descending order and for the same number of repetitions per one functional run (i.e., in one run each numerosity from 1 to 7 was presented 4 times and the baseline of 20 spheres was presented 12 times). This was repeated five times per subject per stimuli condition (i.e., each numerosity was presented 20 times in each stimuli condition of the equal total volume of spheres and equal spheres size).

We extracted from the measured signal of the glove the epochs where spheres were placed in the participant’s hands (Fig. 4a). For each epoch and for each finger we measured the amount of motion, the variation of motion, and the duration of motion. The amount of motion was calculated as a number of peaks of the signal (Fig. 4a) and the variation of motion was the STD of the signal during that period. The duration of motion was calculated between the start and end of the changes in sensor values irrespective of the beginning and end positions. Statistical analysis on the hand motion data was done using JASP (JASP Team(2020). JASP (Version 0.12.2)). All other statistical tests described in this paper were performed in MATLAB.

### Behavioral measures of haptic numerosity perception

Five participants sat behind a curtain with their right hand reaching out. An experimenter placed a different number of spheres (from one to seven) in their hand for 3 s. The experimenter received auditory ques (through headphones the participant could not hear) to mark which numerosity is being tested and when to place or remove the spheres from the participant’s hands. The participants were asked to judge the number of spheres they were exploring and then to click with their left hand on a keyboard, marking the number of spheres they perceived. Here, inter-stimulus period was 4.5 s, starting after the participant had responded. Thus, the participant could respond during, but also after the spheres were taken out of their hands. The spheres were presented in random order. In one test each numerosity was presented 4 times. The total duration of one test was on average 251 s (standard error = 6.5 s). This was repeated 5 times per stimulus condition (i.e., in total each numerosity was presented 20 times in each stimulus condition of equal total volume of spheres and equal individual spheres size). Mean error rate was computed per participant. Reaction time analysis was tested on the median values of each participant.

### Visual numerosity stimuli

Visual stimuli were presented on a 69.84 × 39.29 cm LCD screen (Cambridge Research Systems) at the end of the MRI bore and viewed through a mirror. The total distance from the participant’s eyes was 220 cm. The display resolution was 1920 × 1080 pixels.

Visual numerosity stimuli were similar to previous studies^6,7^. In short, stimuli were generated in MATLAB using PsychToolbox^54,55^. Thin red lines crossed the gray background display and formed a large diagonal cross, aiding fixation at the cross intersection (Fig. 1b). Stimuli were randomly presented groups of black circles of equal size spread roughly homogenously within 2° radius of fixation. The random patterns of circles were presented for 300 ms, and in-between 350 ms of gray background was presented. Each numerosity was presented (in different random patterns) 6 times over 3.9 s before moving to the next. In order to ensure that participants were paying attention, white circles were presented about 10% of the time. Participants were asked to press a button when this happened. Numerosities 1–7 were presented in ascending order, followed by 15.6 s showing 20 circles, followed by 7 to 1 in descending order, followed by a similar presentation of 20 circles for 15.6 s. This cycle was repeated four times during a functional run. No numerosity judgment was required.

### Behavioral measures of visual numerosity perception

Five participants sat in front of a display screen at a distance of 220 cm. We used the same visual stimuli of randomly presented groups of black dots that were presented in the scanner and described above. Thin red lines crossed the gray background display and formed a large diagonal cross, aiding fixation at the cross intersection. Dots were presented for 300 ms followed by a gray background. Here, however, the dots were presented in random order. The participants were asked to judge the number of dots presented and to respond with their left hand, marking on a keyboard how many dots they perceived. The gray background was kept until participants responded (with a minimum display time of 350 ms). In one test each numerosity was presented 5 times. Total duration of one test was on average 98 s (standard error = 2.2 s). This was repeated 4 times, thus in total, each numerosity was presented 20 times. Mean error rate was computed per participant. Reaction time analysis was tested on the median values of each participant.

### MRI acquisition and preprocessing

MRI data were acquired on a 7T Philips Achieva scanner. T1 weighted images were acquired using MP2RAGE sequence with the following parameters: TR = 6.8 ms; TE = 2.3 ms, flip angle = 5°; isotropic resolution of 0.8^3^, SENSE factor = 2; slices = 205. Functional runs were acquired using a 32 channel head coil with the following parameters: isotropic resolution of 1.75 mm^3^; TR/TE = 1950/25 and TR/TE = 1500/25 for visual and haptic numerosity functional scans, respectively; flip angle = 70; multiband factor = 2. The haptic data included 52 slices and the visual data 64 slices. The haptic numerosity runs included 208 TRs and lasted 312 s. The visual numerosity runs included 182 TRs and lasted for 354.9 s.

The numerosity experiments, either visual or haptic, included 4–8 repeated runs in each scanning session. Each stimulus configuration (visual, haptic—equal individual size and equal total volume) was acquired in 1 or 2 scanning sessions on different days. In general, the visual numerosity condition included 8 functional repetitions, and the haptic numerosity included 8 or 16 repetitions per condition.

T1-wieghted anatomical scans were resampled to an isotropic resolution of 1 × 1 × 1 mm and then preprocessed and segmented using cbs-tools^56^. Segmentation errors were hand-edited using ITK-SNAP^57^. The gray matter cortical surface was reconstructed and rendered as a smooth 3D surface^58^.

Functional runs were corrected for head movement and motion using AFNI. The first 6 timeframes of the visual numerosity functional scans scan were discarded to ensure steady-state magnetization. The first eight timeframes were discarded from the haptic numerosity functional scans. Using Vistasoft (https://github.com/vistalab/vistasoft/wiki) the anatomical images were registered to the functional runs. The time-series data were aligned to the T1-weighted anatomical space and then averaged based on each functional condition (visual, haptic equal individual sphere size, haptic equal total spheres volume). Data were interpolated to the anatomical segmentation space using trilinear interpolation. To increase signal strength, data from all recording points (voxels) were collapsed and averaged onto the nearest point on the cortical surface. This formed a (folded) two-dimensional representation of the gray matter nodes and increased signal strength^6^. pRF modeling and subsequent statistical analyses were done at this space. The statistics were adjusted to account for upsampling from the acquired data to the anatomical space. No spatial or temporal smoothing was applied to the functional data.

### fMRI data analysis

pRF modeling was applied to estimate numerosity responses^6,7,59^. Briefly, the pRF model describes the averaged tuning of the underlying neural populations using logarithmic Gaussian functions that are characterized by preferred numerosity (mean of the Gaussian) and tuning width (STD of the Gaussian).

At each gray matter voxel, the pRF model is estimated based on the fMRI data and the time course of presented numerosities. For each candidate preferred numerosity and tuning width, a predicted neural response time course is calculated as the amplitude of the candidate neural response function at each time point’s presented numerosity. Each candidate predicted neural response time course is then convolved with the haemodynamic response function (HRF) to create a candidate predicted fMRI time course. The chosen pRF parameters for each voxel are those whose predicted fMRI time course is best correlated with the voxel’s measured fMRI time course. Last, participant specific HRF parameters were estimated over the whole fMRI volume^60^. These parameters were used to refit the pRF.

The pRF fitting procedure allows preferred numerosity estimates outside the range of the presented stimuli, ensuring estimates within the stimulus range are not just the best of a limited set. We excluded from analysis any recording sites where the preferred numerosity was outside our presented range.

To convert the variance explained (*R*^2^) measures to probabilities of observing these model fits by chance, we built a null distribution using the same model-fitting procedure on 225,744 white matter voxels from the same scans. For each variance explained we calculated the proportion of these voxels with model fits exceeding that value. Subsequent analyses included only gray matter voxels where the variance explained of the pRF model was higher than 30% (equivalent to a probability of 0.016 of observing this goodness of fit by chance).

### Cross validation

In order eliminate recording sites where neural activity may be better explained by the changes in the presence of stimuli in the hand and their haptic exploration (“on–off” stimuli presentation) rather than tuning to stimulus numerosity, we split the data of each participant into two. The data from both haptic conditions (equal total volume and equal individual spheres size) was split based on even-odd runs and included between 8 and 16 runs. We then fitted on each split data a general linear model (GLM) where the timing of stimulus presentation served as a predictor. We also fitted the numerosity pRF model on each half-split of data. Then, the goodness of fit of the pRF prediction from one half-split was evaluated on the time course of the other half, yielding new values of goodness of fit. Voxels, where the variance explained by the GLM “on–off” model was higher than the cross-validated pRF numerosity models, were excluded from further analysis.

### Definition of region of interest

Haptic numerosity data: We rendered the preferred numerosities of the response model from the average of the two stimuli condition onto the cortical surface. We excluded recording sites where the variance explained was lower than 30%, numerosity preference was outside stimuli range, or the fMRI time course was better explained by an “on–off” response to stimuli presentation. This presentation showed similar regions and topographic maps of numerosity-selective responses as were previously reported for equivalent visual numerosity stimuli^7^. In each of these regions of interest (ROI), we manually defined lines on the lowest and highest points of preferred numerosity (“end” borders). The edges of the map (“side” borders) were defined around these local regions.

Visual numerosity data: We rendered the preferred numerosities of the response model onto the cortical surface. We excluded recording sites where the variance explained was lower than 30% or numerosity preference was outside the stimuli range. Again, this presentation showed similar regions and topographic maps of numerosity-selective responses to those previously reported^7^. We manually defined the borders of these maps.

### Analysis of change across the haptic ROIs

In each ROI, for each recording site, we calculated the cortical surface distances to the nearest points on the two “end” borders of the map. The ratio between the two distances gives a normalized distance along the ROI, in the main direction of change in numerosity preferences. The ratio was then multiplied by the mean length of the ROI in the same direction.

We then binned the recording sites within every 2 mm interval along the main direction of change in the map. The mean and standard error of the numerosity preferences within each bin was calculated. We fitted logarithmic functions to bootstrapped samples of the bin means. From these bootstrapped fits we took the median slope and intercept as the best fitting numerosity progression. 95% confidence interval was determined by finding the 2.5 and 97.5 percentiles of all the bootstrapped lines. The statistical significance of the slopes was determined with permutation analysis, where the order of distance bins was randomized (10,000 times). Slopes were fitted at each permutation, and the probability of finding the observed slope by chance was calculated as the number of times where the slope in the randomized permutation analysis was equal to or greater than the observed slope.

We calculated the significance of the tuning width change with preferred numerosity in a similar way, where the recording points were binned within every 0.25 increase in preferred numerosity. The mean of the tuning width within each bin was calculated. For the group analysis, we calculated the average and standard error of the bins across participants. Linear functions were fitted to bootstrapped samples of the bin means, where the best-fitting tuning width progression was the median slope and intercept. Similar permutation analysis, as described above, was used to calculate the probability of finding the observed tuning width change by chance.

We assessed the differences in cortical surface area preferring specific numerosity range. We grouped the preferred numerosity across participants and hemispheres. Each map was tested for a trend of linear decrease along the range of numerosities (1–7) using a planned comparison contrast. We corrected for the number of tests using false discovery rate.

### Correlation and overlap analysis between haptic and visual numerosity preferences

The percentage of overlap between the haptic and visual numerosity maps was calculated for cortical points that suppress a threshold of variance explained above 30% in both modalities. The percentage of shared area was calculated both relative to the visual numerosity maps and haptic numerosity maps.

One-way ANOVA followed by post hoc analysis using Bonferroni correction for multiple comparisons was performed in order to test any differences in the percentage of the shared area between the maps (averaged across hemispheres). Before the statistical tests the percentage values were transformed using arcsin transformation.

Pearson correlation analysis was run in the same recording sites that were shared between the maps of the haptic and visual modalities (for each map and each participant, see Supplementary Figs. S13 and 14). Pearson correlation analysis was also run on the shared recording sites between the maps of the two haptic conditions that exceeded variance explained of 30% in both conditions.

We ran a test-retest on the visual numerosity maps. We split the visual numerosity data into two based on even-odd runs (4 runs were included in each half-split of data). We then fitted the numerosity pRF model on each half-split of data. Pearson correlation analysis was run between the preferred numerosities of the two visual maps in the recording sites of the visual numerosity maps (that exceeded variance explained of 30%).

Before the statistical tests on the correlation coefficients in the shared areas between and within modalities, the correlation coefficients were transformed into Z distribution using Fisher Z-transformation.

### Conversion to MNI coordinates

Analyses were performed in the participant’s native space. In order to localize the average position of the haptic numerosity maps across participants we transformed the centre of the maps of each participant into MNI space using MINC toolkit (http://packages.bic.mni.mcgill.ca), applying rigid alignment and linear scaling^36^. We then averaged the resulting MNI coordinates across participants.

In order to show the overlap of the maps across participants (Fig. 3a) we also transformed each participant’s anatomical MRI data, together with the map surfaces, centers, and “end” borders to the Talairach N27 surface^4^. This was done using AFNI’s 3dAllineate and 3dNwarpApply tools.

### Statistics and reproducibility

The visual numerosity experiment is a replication of former studies^5–7,61^, showing similar results across studies. The haptic numerosity experiment includes two independent conditions that revealed similar results (see Supplementary Figs. 3, 4, 7, and 8). The numerosity perception studies are replicating many previous studies^25,38,62^.

### Reporting summary

Further information on research design is available in the Nature Research Reporting Summary linked to this article.

## Supplementary information

Supplementary Information

Reporting Summary

## Data Availability

The data sets generated during the current study are available from the corresponding author upon reasonable request. Source data are provided with this paper.

## Source data

Source Data

## Code availability

The code that supports the findings of this study is available from the corresponding author upon reasonable request.
