# Supplementary material for: Topographic maps representing haptic numerosity reveals distinct sensory representations in supramodal networks
Source: Nat Commun. 2021 Jan 11;12:221. doi: 10.1038/s41467-020-20567-5 (PMC7801743; doi:10.1038/s41467-020-20567-5)
Supplement: Supplementary file 1 — Supplementary Information [file 41467_2020_20567_MOESM1_ESM.pdf]

Topographic maps representing haptic numerosity reveals distinct sensory representations in supramodal networks

Supplementary material

Shir Hofstetter[1], Yuxuan Cai [1,2], Ben M. Harvey[3], Serge O. Dumoulin[1,2,3]

1. Spinoza Centre for Neuroimaging, 1105 BK Amsterdam, The Netherlands
2. Department of Experimental and Applied Psychology, VU University Amsterdam, 1181 BT Amsterdam, The Netherlands
3. Department of Experimental Psychology, Helmholtz Institute, Utrecht University, 3584 CS Utrecht, The Netherlands

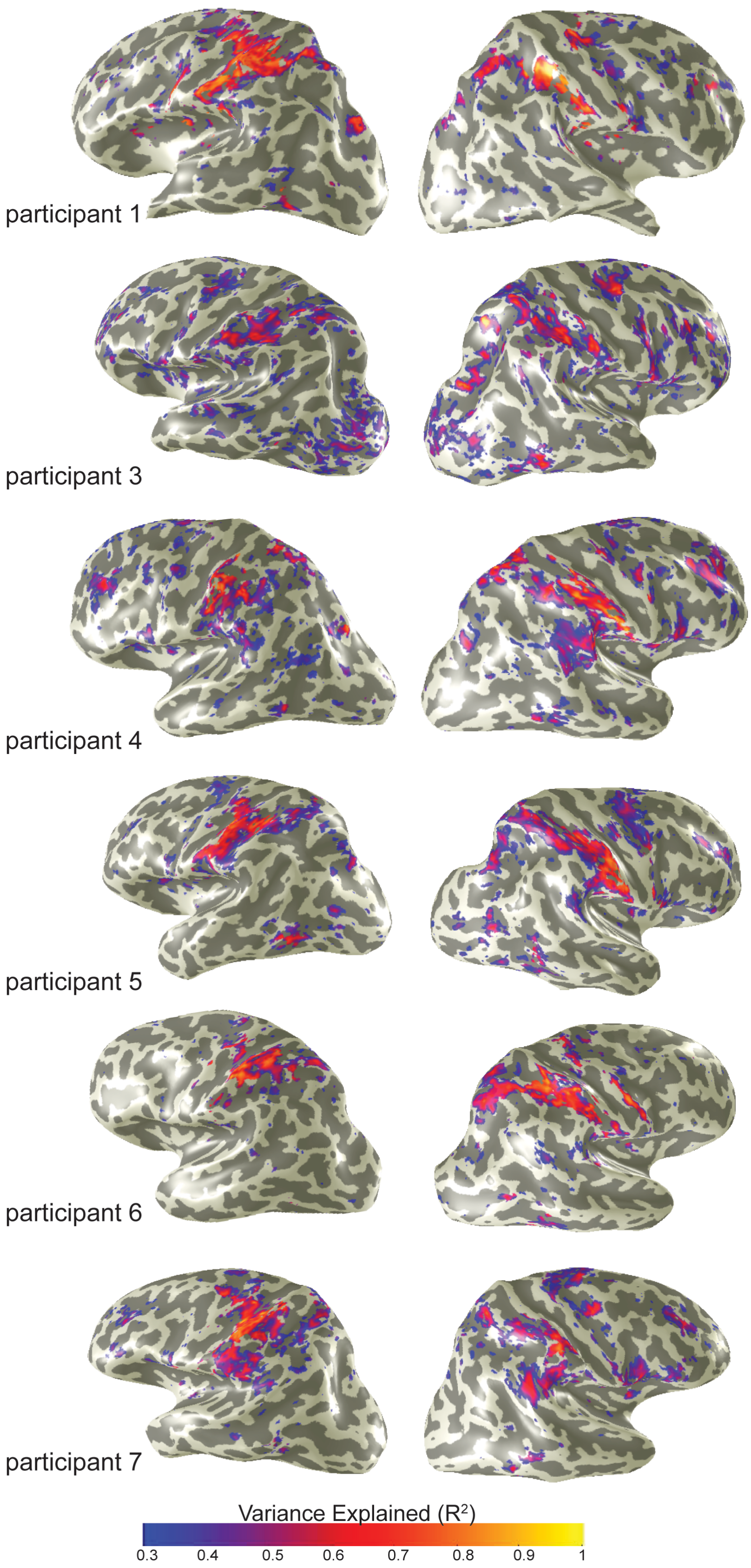

Supplementary figure 1: Goodness of fit of numerosity-selective neural models averaged across stimulus conditions (variance explained >30%). The maps show the goodness of fit to numerosity-selective responses after removal of areas which exhibit general movement responses that are unselective to numerosity (see methods).

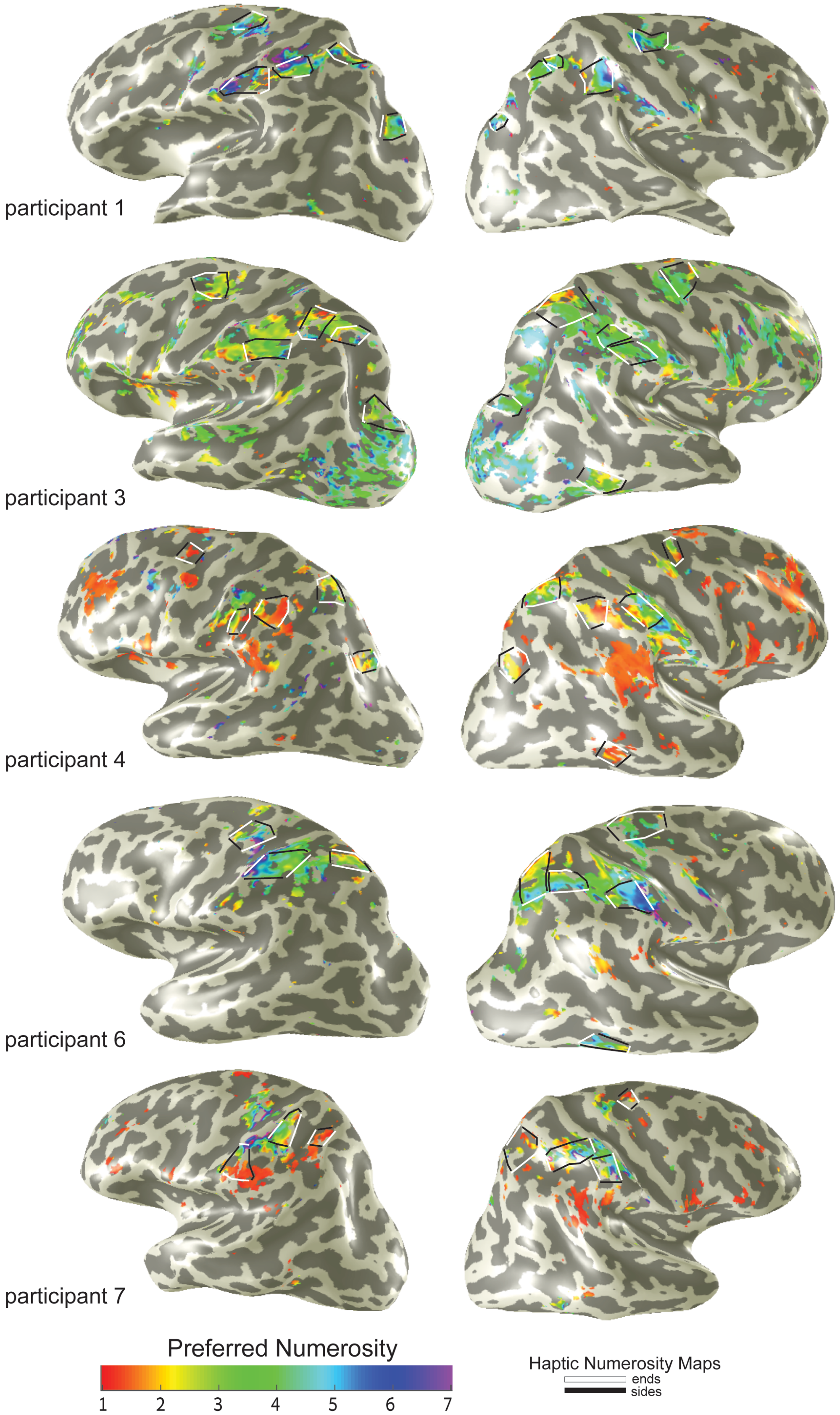

Supplementary figure 2: Numerosity preferences for data averaged from both haptic stimulus conditions (i.e., equal total volume and equal individual sphere size; variance explained > 30%). Colors represent preferred numerosity. The borders of lowest to highest preferred numerosity in each map are marked by white lines. Black lines complete the margins of the maps.

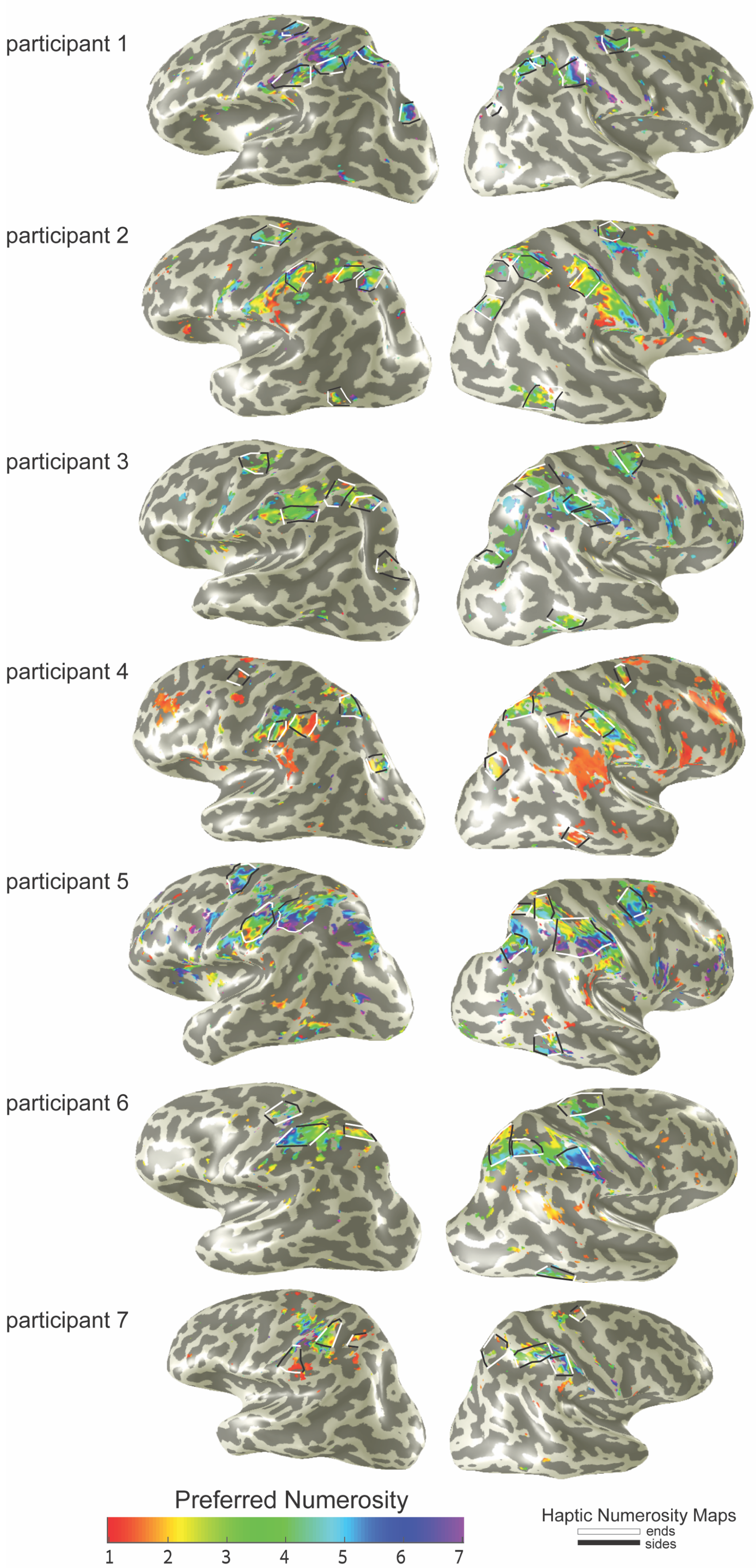

Supplementary figure 3: Numerosity preferences for data in the haptic stimulus condition of equal total volume of spheres (variance explained > 30%). Colors represent preferred numerosity. White and black lines show the outline of the haptic numerosity maps from data averaged from both haptic stimulus conditions.

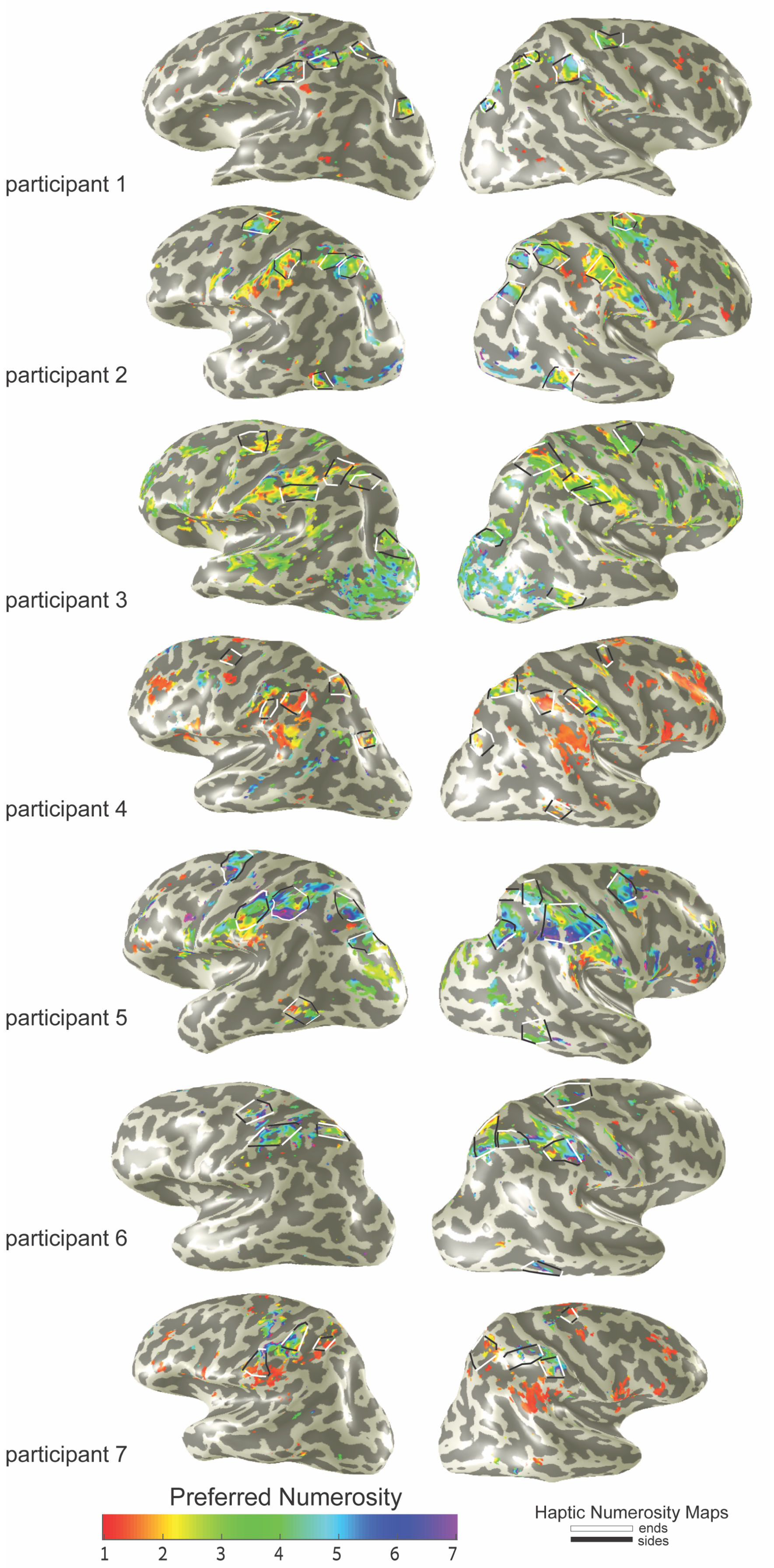

Supplementary figure 4: Numerosity preferences for data in the haptic stimulus condition of equal individual sphere size (variance explained > 30%). Colors represent preferred numerosity. White and black lines show the outline of the haptic numerosity maps from data averaged from both haptic stimulus conditions.

Supplementary table 1: Maps centres positions in Montreal Neurological Institute (MNI) space. Values are given as mean (SD). Source data are provided as a source data file.

	Left Hemisphere				Right Hemisphere			
	Number of maps	x	y	z	Number of maps	x	y	z
NhTO	2	-55(4)	-61(1)	-13(6)	5	53 (4)	-59(4)	-11(6)
NhPO	4	-26(2)	-83(6)	34(3)	5	24(4)	-81(7)	35(8)
NhPC1	7	-24(4)	-60(6)	63(8)	6	19(5)	-66(7)	66(8)
NhPC2	7	-38(6)	-44(6)	59(6)	7	32(4)	-50(8)	60(10)
NhPC3	6	-46(7)	-31(5)	45(8)	7	43(8)	-35(7)	49(3)
NhF	6	-24(5)	-7(7)	61(8)	7	22(2)	-11(7)	61(7)

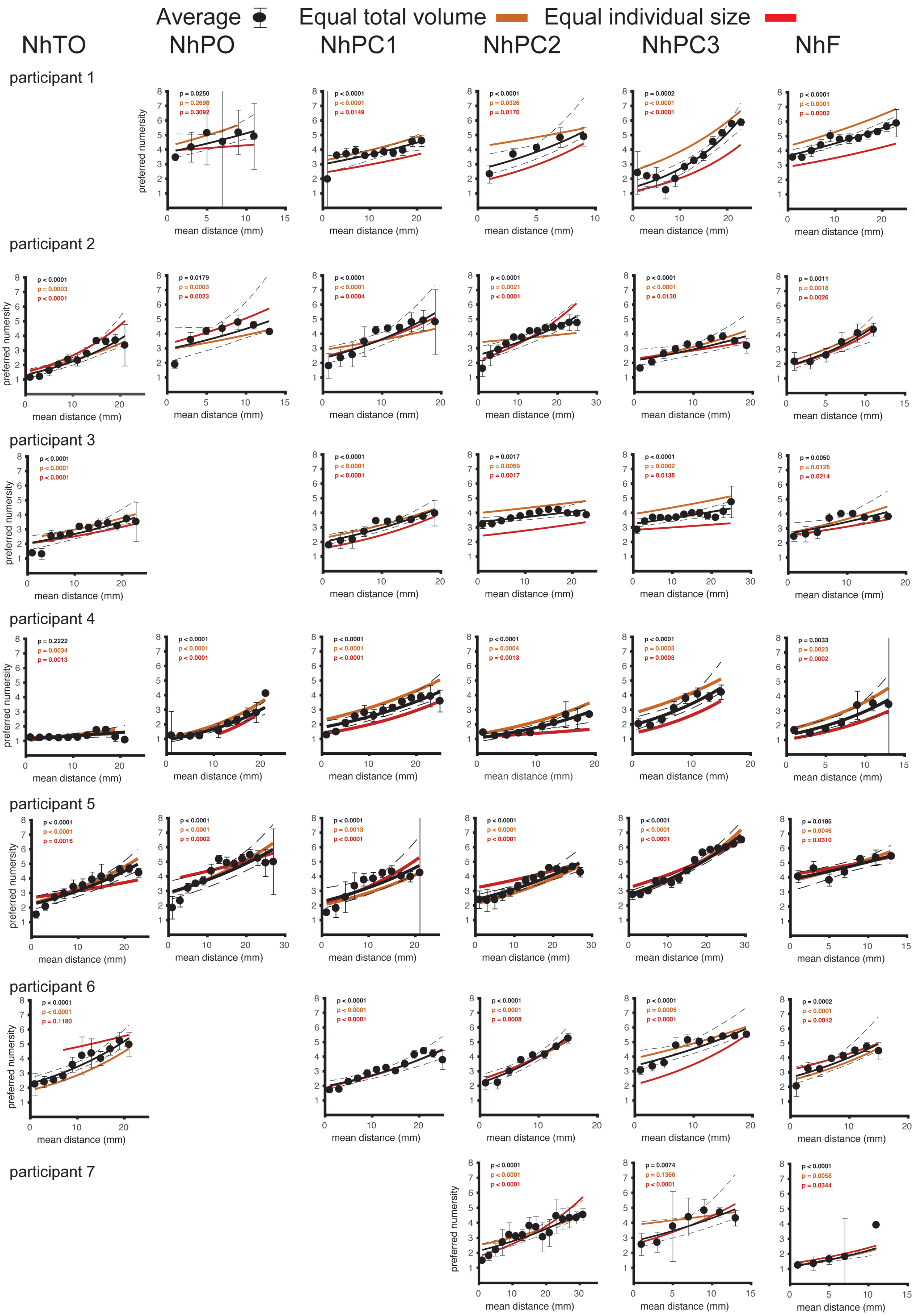

Average
Equal total volume
Equal individual size
NhTO
NhPO
NhPC1
NhPC2
NhPC3
NhF
participant 1
participant 2
participant 3
participant 4
participant 5
participant 6
participant 7
preferred numersity
mean distance (mm)

Supplementary figure 5:

Preferred numerosities of the maps in the right hemisphere plotted as a function of the distance along cortical surfaces (measured between the white lines, see supplementary figure 2). Solid lines show logarithmic fits of stimulus configuration and their mean response. Dashed lines show 95% confidence interval of the logarithmic fit of the responses averaged across stimulus configurations, determined by bootstrapping. Colored text gives the probability of the observed change from permutation analysis. Error bars show the standard error of the mean for each data point. The number of recording sites across the data points, averaged across maps for each participant: participant 1- n(min)=4, n(max)=18, n(mean)=29; participant 2- n(min)=8, n(max)=35, n(mean)=22; participant 3- n(min)=8, n(max)=49, n(mean)=29; participant 4- n(min)=5, n(max)=31, n(mean)=18; participant 5- n(min)=12, n(max)=55, n(mean)=38; participant 6- n(min)=8, n(max)=42, n(mean)=25; participant 7- n(min)=5, n(max)=29, n(mean)=14

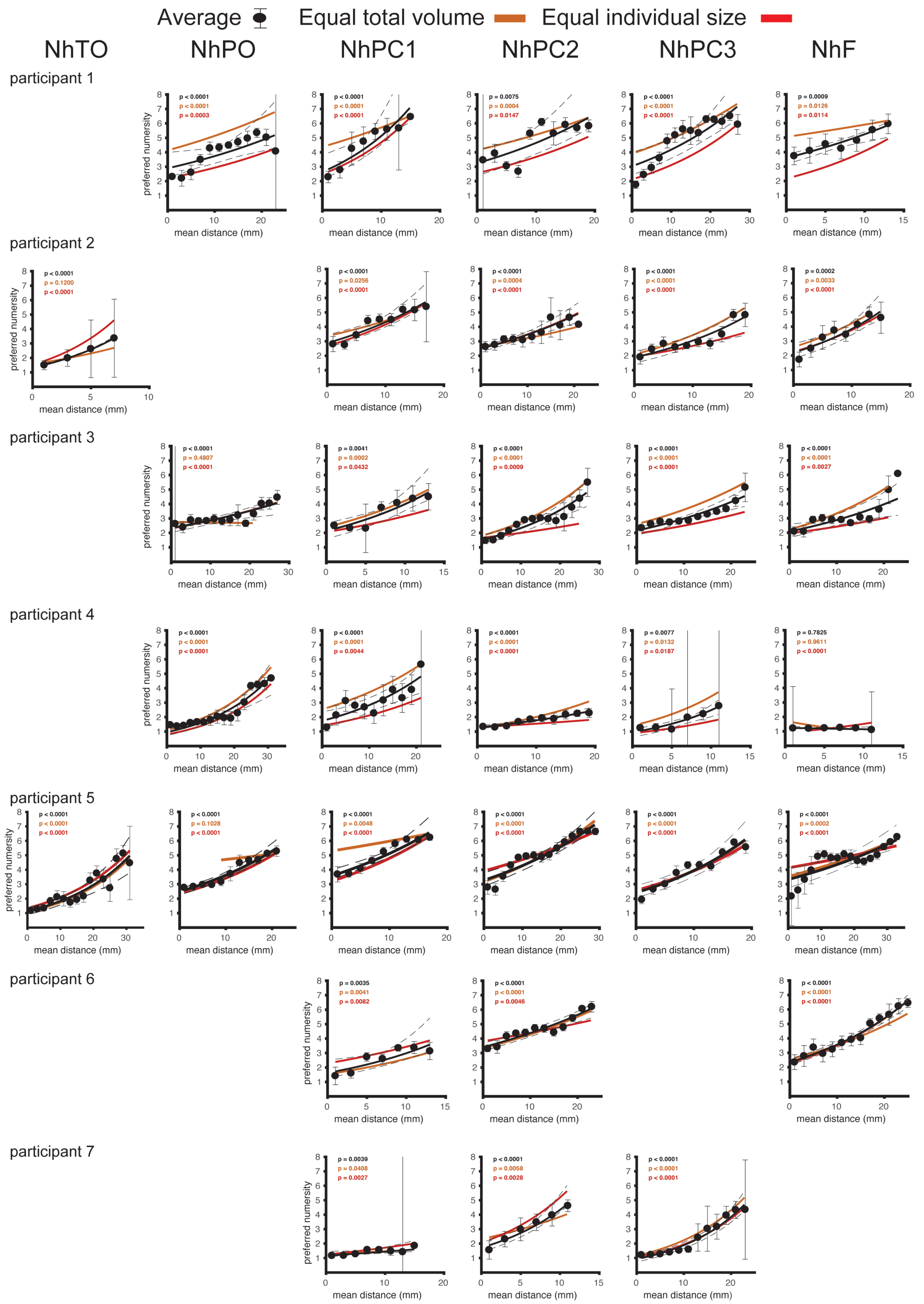

Average
Equal total volume
Equal individual size
NhTO
NhPO
NhPC1
NhPC2
NhPC3
NhF
participant 1
participant 2
participant 3
participant 4
participant 5
participant 6
participant 7
preferred numersity
mean distance (mm)

Supplementary figure 6:

Preferred numerosities of the maps in the left hemisphere plotted as a function of the distance along cortical surfaces (measured between the white lines, see supplementary figure 2). Solid lines show logarithmic fits of stimulus configuration and their mean response. Dashed lines show 95% confidence interval of the logarithmic fit of the responses averaged across stimulus configurations, determined by bootstrapping. Colored text gives the probability of the observed change from permutation analysis. Error bars show the standard error of the mean for each data point. The number of recording sites across the data points, averaged across maps for each participant: participant 1- n(min)=3, n(max)=27, n(mean)=15; participant 2- n(min)=4, n(max)=19, n(mean)=34; participant 3- n(min)=6, n(max)=40, n(mean)=24; participant 4- n(min)=4, n(max)=23, n(mean)=13; participant 5- n(min)=10, n(max)=60, n(mean)=35; participant 6- n(min)=5, n(max)=37, n(mean)=22; participant 7- n(min)=1, n(max)=28, n(mean)=14.

Supplementary table2: The frequency of significant preferred numerosity progressions by numerosity map and participant. The number of maps (n) are shown per category and include maps of the two stimulus configuration and their average (i.e., maximum number of maps per participant in each ROI is 3). Source data are provided as a source data file.

**Left Hemisphere**				**Right Hemisphere**			
**By map**		**By participant**		**By map**		**By participant**	
NhT (n=6)	83%	Participant 1 (n=15)	100%	NhTO (n=15)	87%	Participant 1 (n=15)	87%
NhPO (n=12)	83%	Participant 2 (n=15)	93%	NhPO (n=12)	83%	Participant 2 (n=18)	100%
NhPC1 (n=21)	100%	Participant 3 (n=15)	93%	NhPC1 (n=21)	95%	Participant 3 (n=15)	100%
NhPC2 (n=21)	100%	Participant 4 (n=15)	87%	NhPC2 (n=21)	100%	Participant 4 (n=18)	94%
NhPC3 (n=18)	100%	Participant 5 (n=18)	94%	NhPC3 (n=21)	95%	Participant 5 (n=18)	100%
NhF (n=18)	89%	Participant 6 (n=9)	100%	NhF (n=21)	100%	Participant 6 (n=15)	93%
		Participant 7 (n=9)	100%			Participant 7 (n=12)	83%

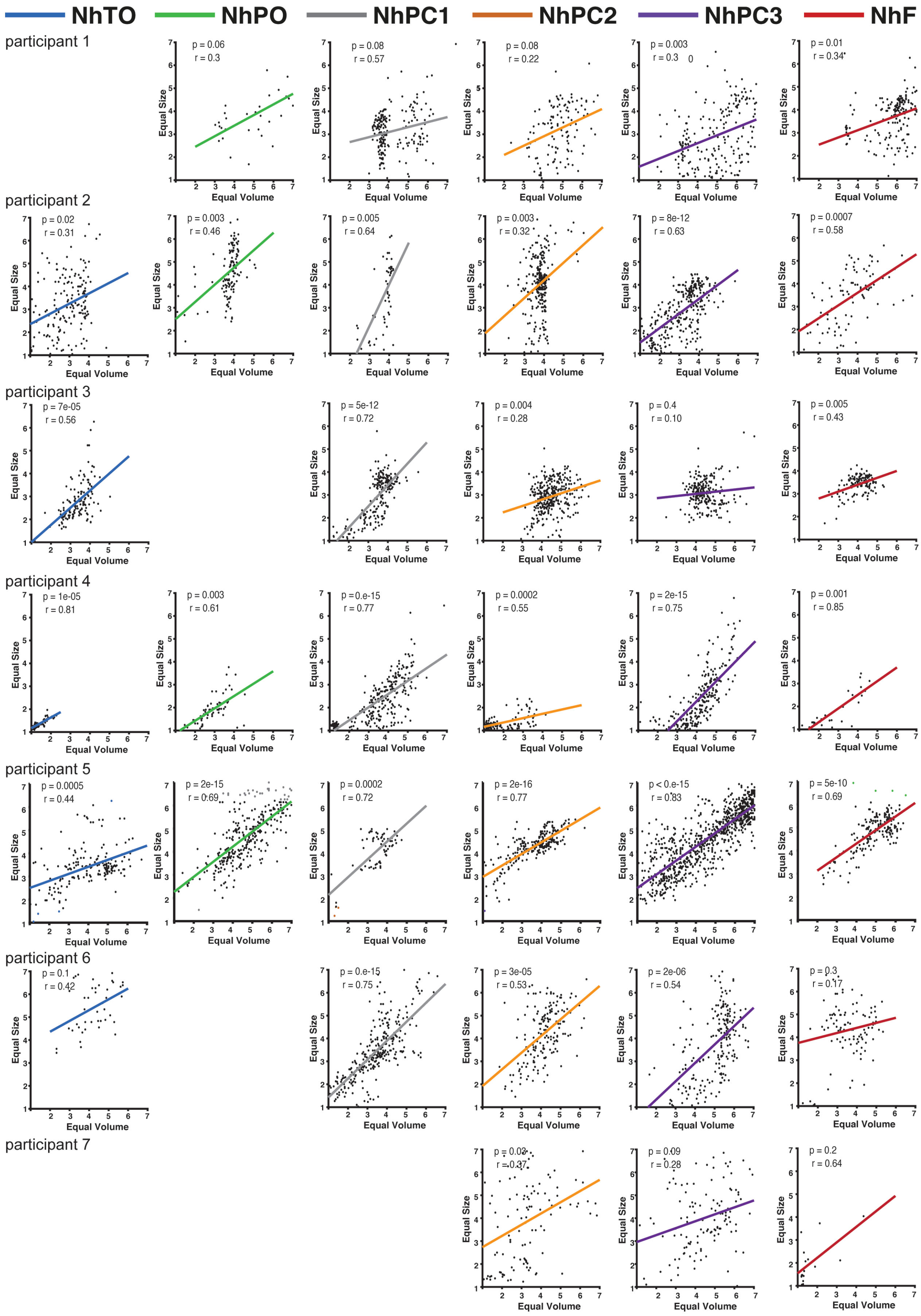

Supplementary figure 7:

Pearson correlation analyses between preferred numerosity of the two haptic stimulus configurations (i.e., equal total volume and equal individual sphere size) in maps of the right hemisphere.

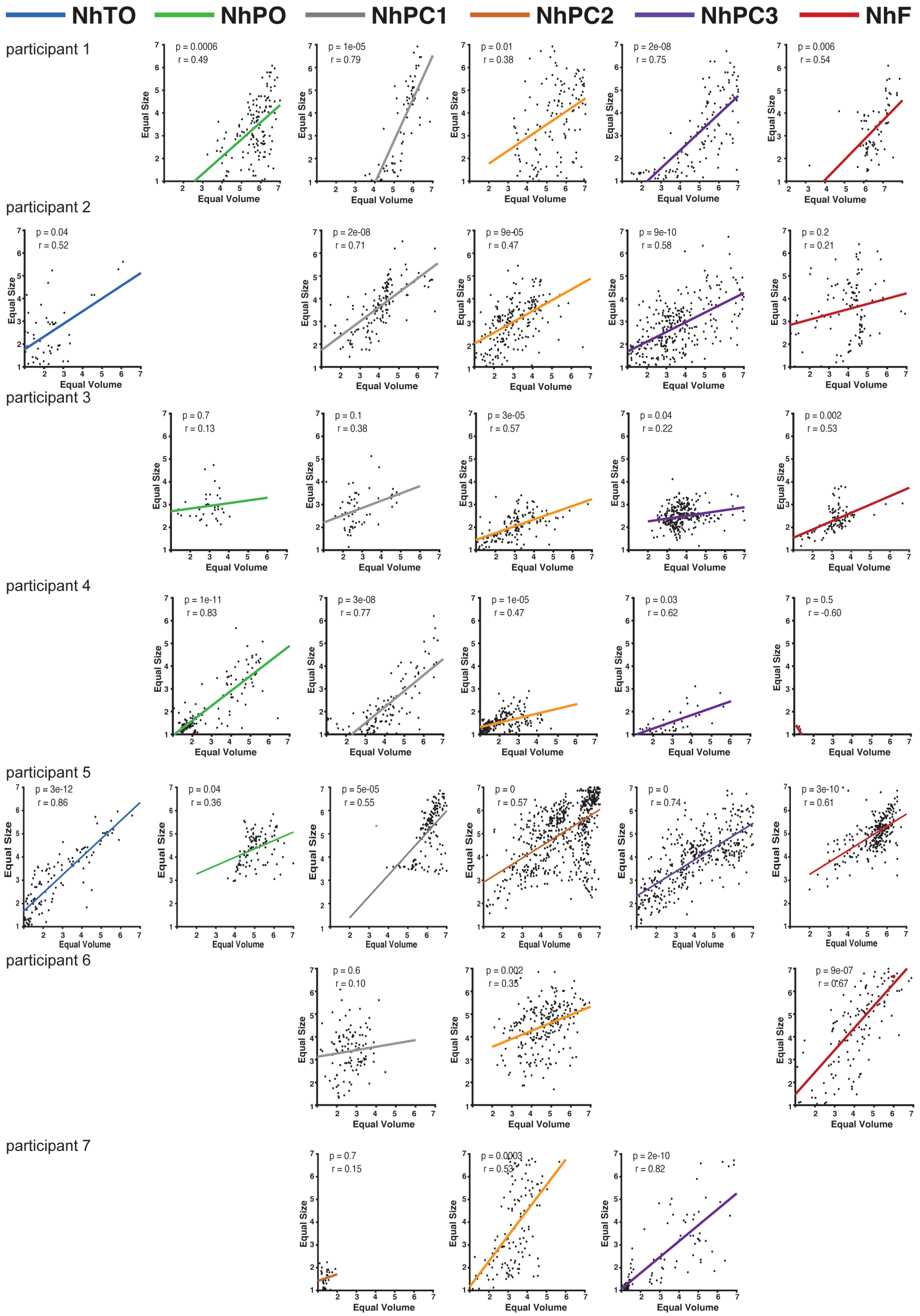

Supplementary figure 8:

Pearson correlation analyses between preferred numerosity of the two stimulus configurations (i.e., equal total volume and equal individual sphere size) in maps of the left hemisphere.

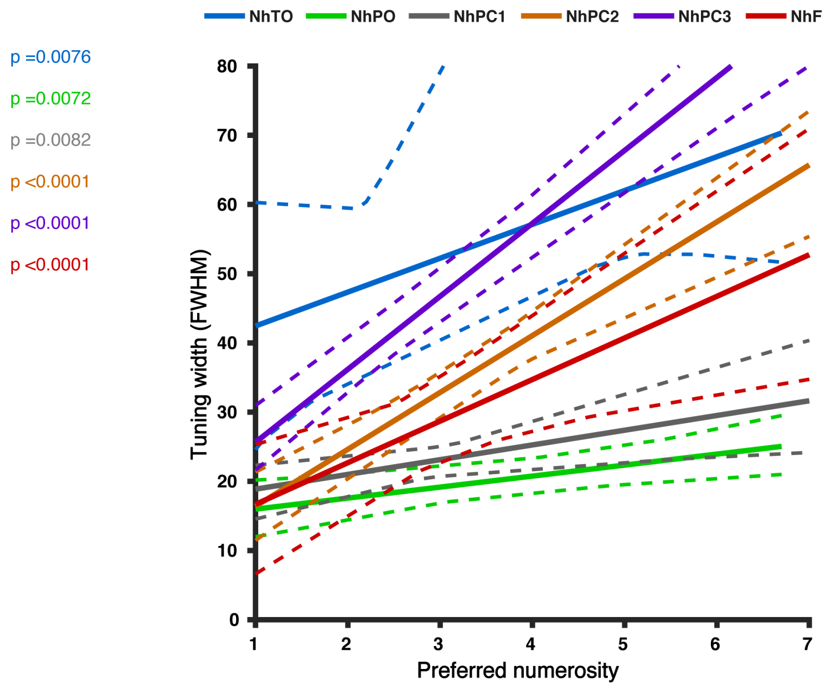

Supplementary figure 9:

Progression of population tuning widths with preferred numerosity averaged across participants in the left hemisphere. Progression of tuning widths of preferred numerosity was fitted with a linear function (solid lines) with 95% confidence intervals to the fit (dashed lines) determined by bootstrapping. The text gives the probability of the observed change from permutation analysis for each map.

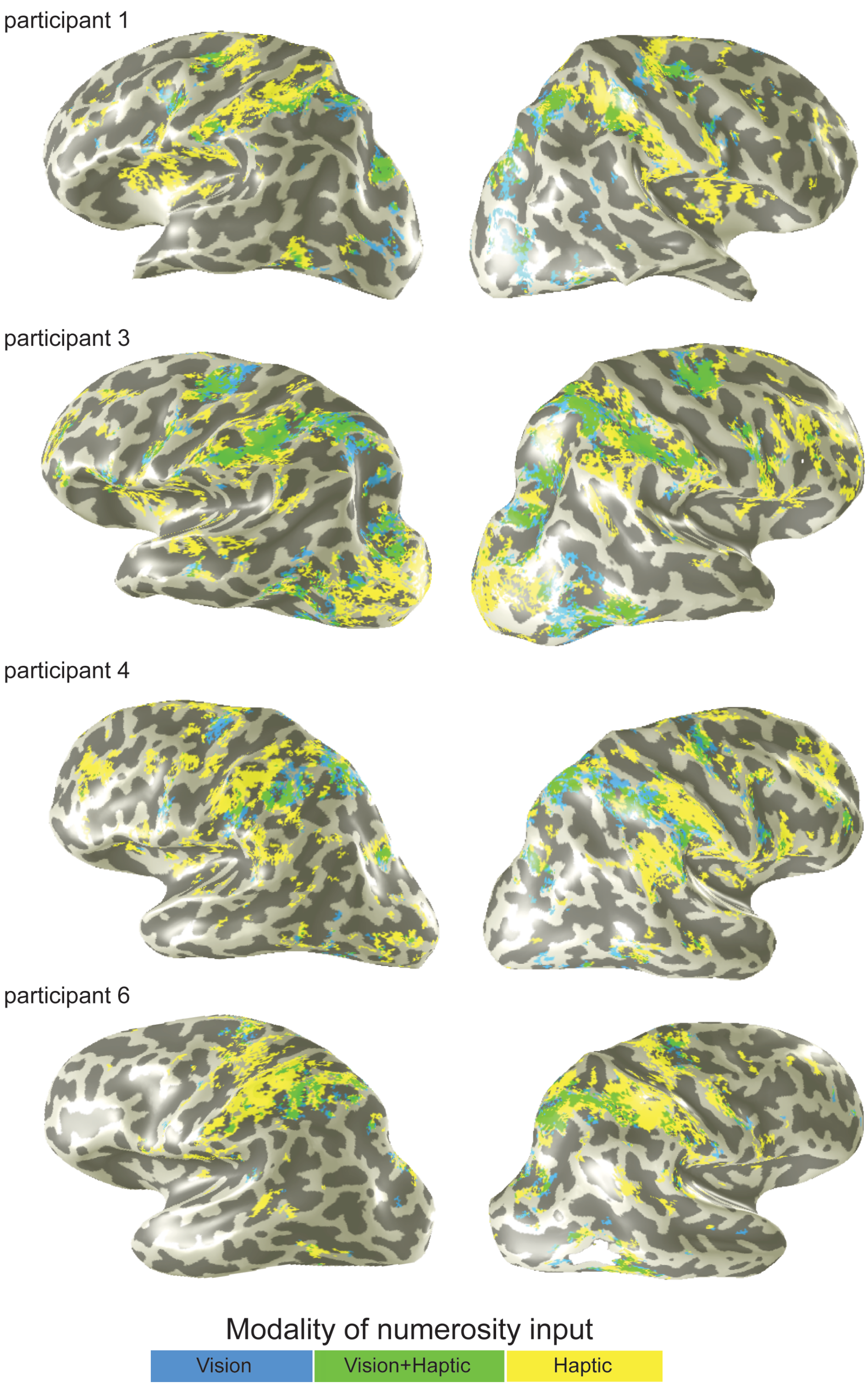

Supplementary figure 10:

Maps of neural responses to numerosity across sensory modalities. Colors represent which type of sensory input produced numerosity-selective neural response (as captured by the pRF model with a variance explained >30%).

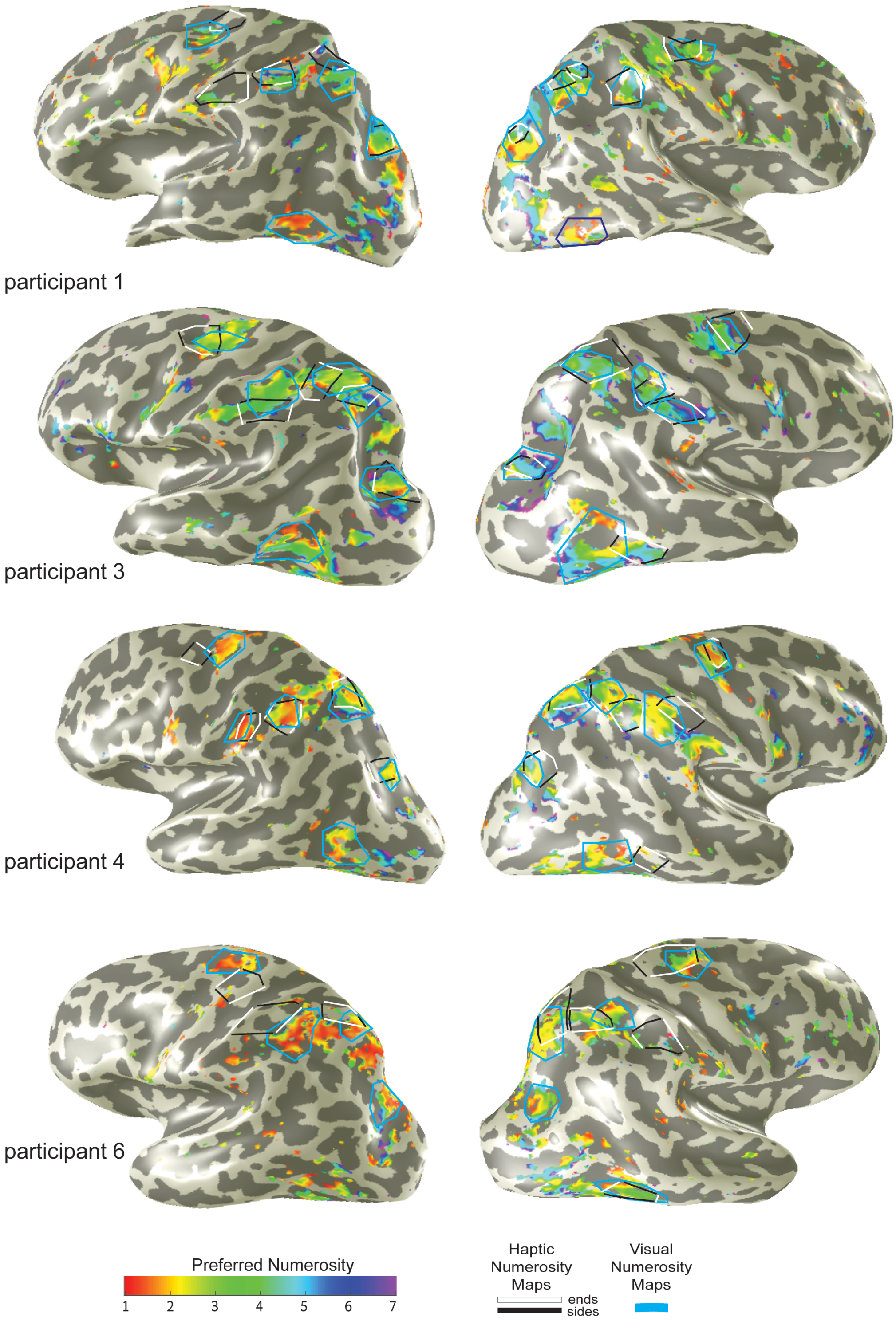

Supplementary figure 11:

Maps of preferred visual numerosity overlaid with the outlines of the visual numerosity maps (blue) and the outlines of the haptic numerosity maps (white lines mark the borders of lowest to highest preferred numerosity for each haptic map, black lines complete the margins of the maps; these are similar to the borders overlaid in supplementary figure 2).

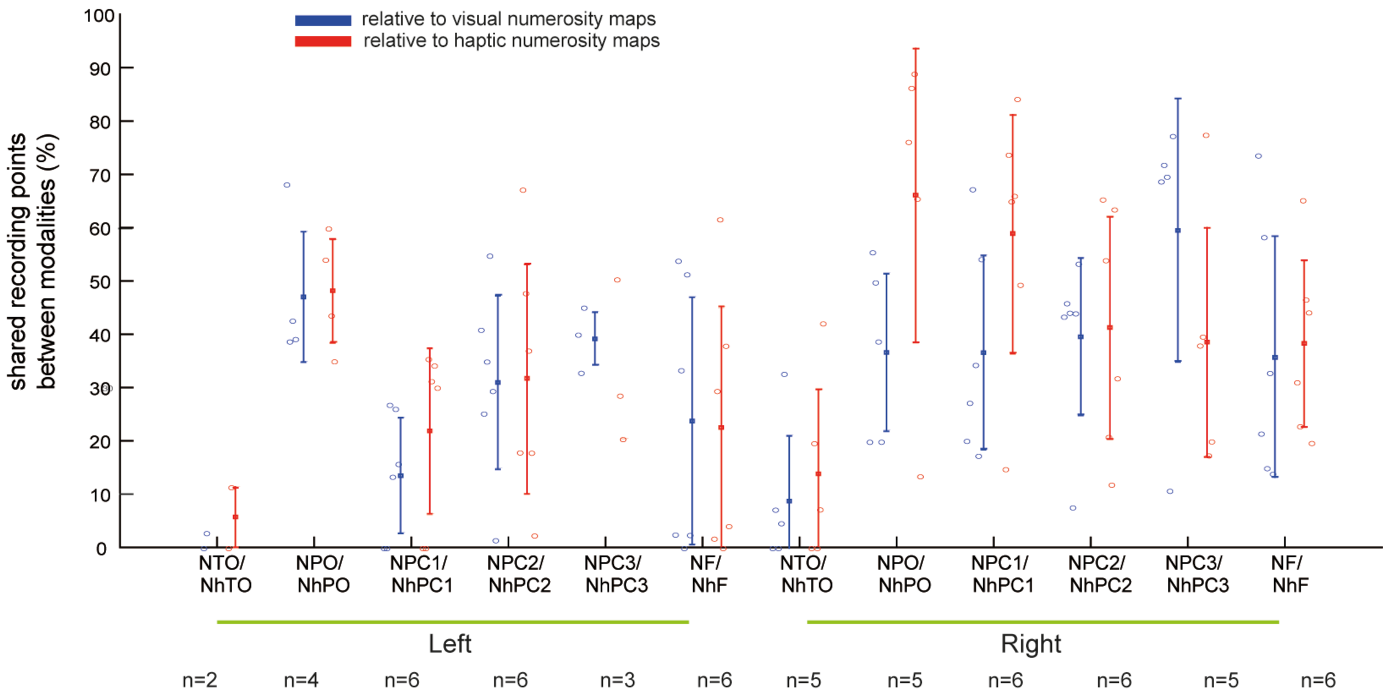

Supplementary figure 12: Percentage of the number of cortical points shared between the visual and haptic numerosity maps relative to the size of the maps. Error bars plots show the mean percentage of shared cortical points averaged across subjects (square) with the corresponding standard deviation of the mean. Individual subjects are represented by dots.

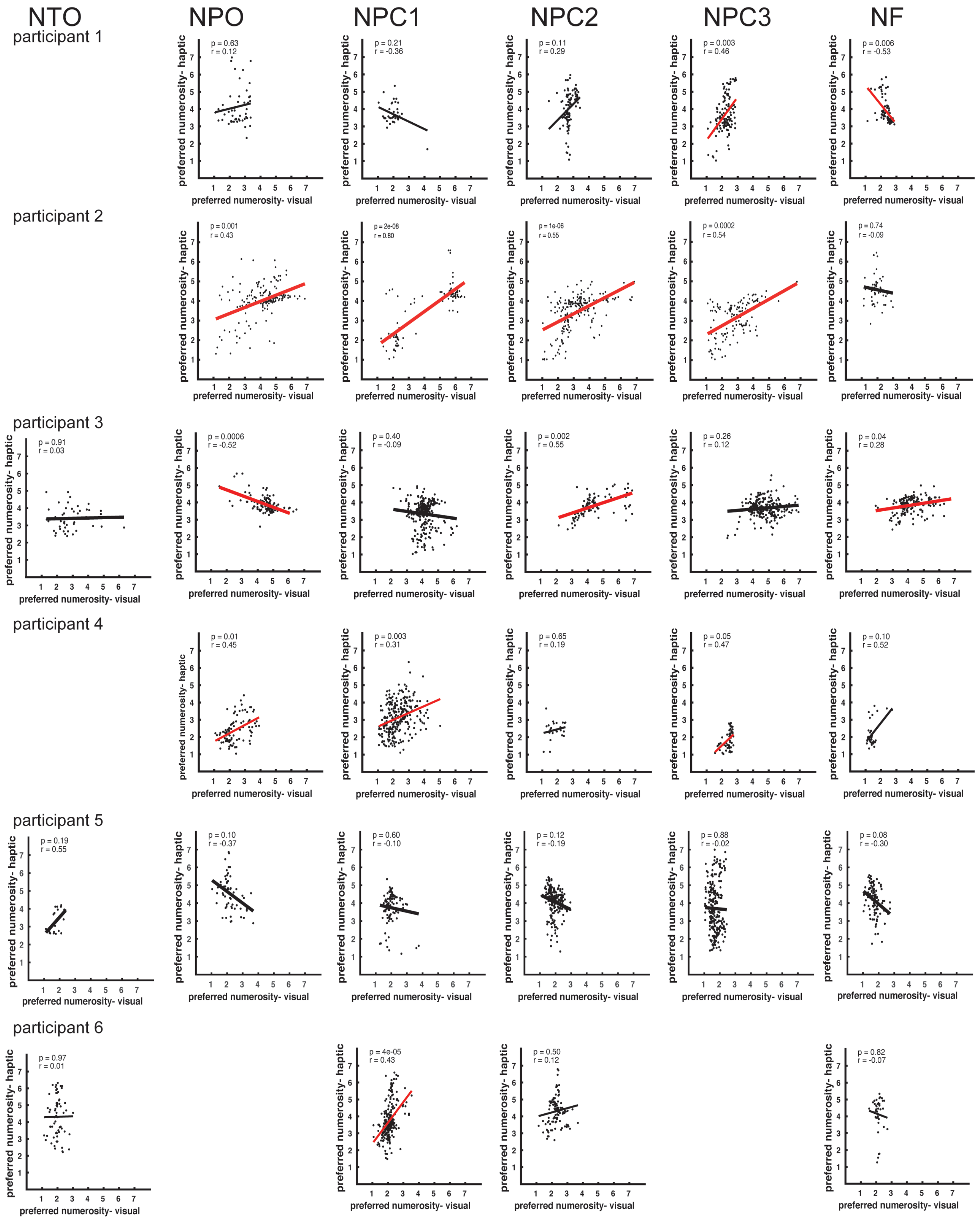

Supplementary figure 13:

Pearson correlation analyses between preferred numerosity of the visual and haptic maps in the right hemisphere (variance explained > 30% in both modalities). Red lines indicate significant correlations.

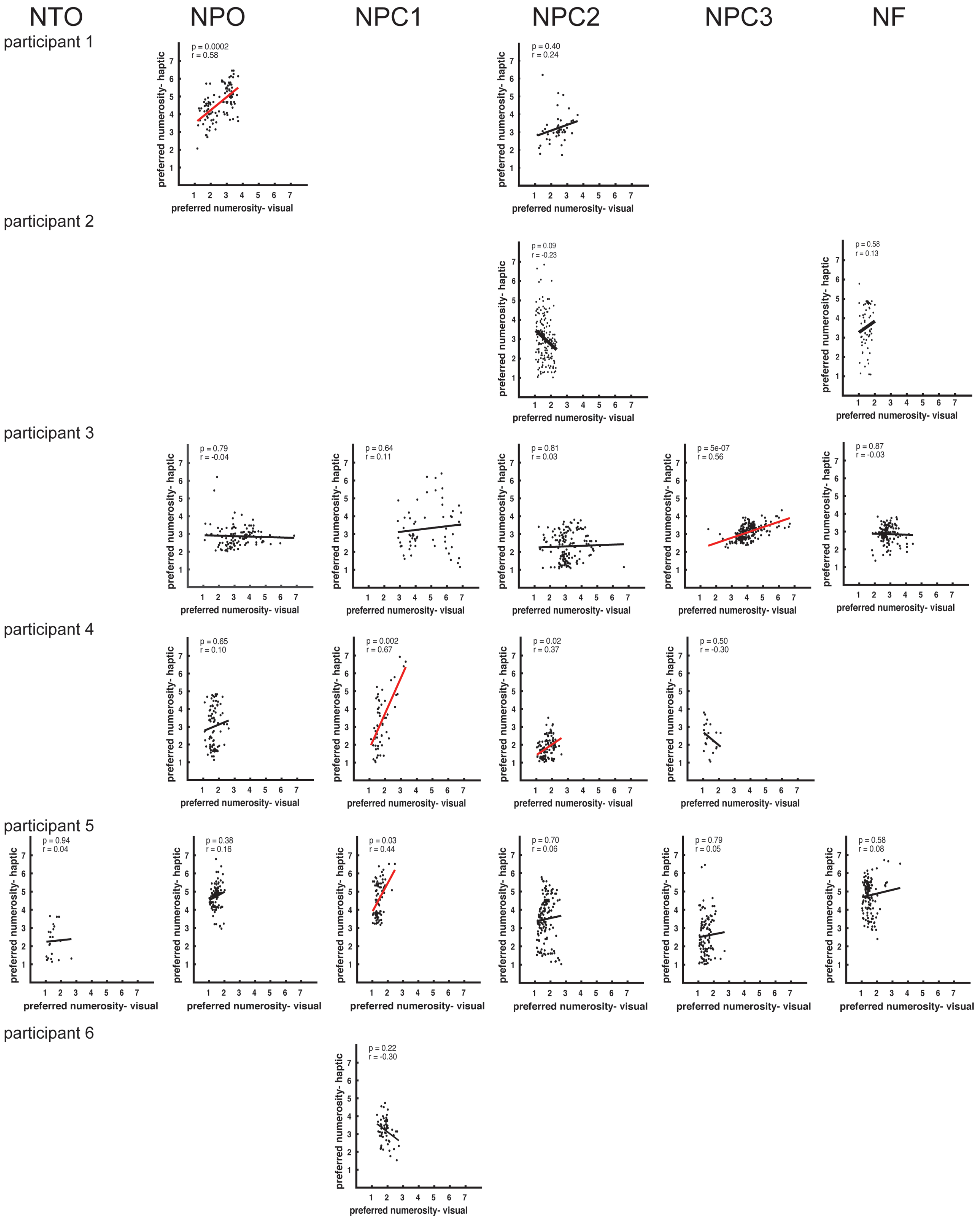

Supplementary figure 14:

Pearson correlation analyses between preferred numerosity of the visual and haptic maps in the left hemisphere (variance explained > 30% in both modalities). Red lines indicate significant correlations.
