# Supplementary material for: Topographic maps representing haptic numerosity reveals distinct sensory representations in supramodal networks
Source: Nat Commun. 2021 Jan 11;12:221. doi: 10.1038/s41467-020-20567-5 (PMC7801743; doi:10.1038/s41467-020-20567-5)
Supplement: Supplementary file 2 — Reporting Summary [file 41467_2020_20567_MOESM2_ESM.pdf]

nature research

Corresponding author(s): Shir Hofstetter
Last updated by author(s): 11/16/2020

Reporting Summary

Nature Research wishes to improve the reproducibility of the work that we publish. This form provides structure for consistency and transparency in reporting. For further information on Nature Research policies, see our Editorial Policies and the Editorial Policy Checklist.

Statistics

For all statistical analyses, confirm that the following items are present in the figure legend, table legend, main text, or Methods section.

n/a	Confirmed	
☐	☒	The exact sample size (*n*) for each experimental group/condition, given as a discrete number and unit of measurement
☐	☒	A statement on whether measurements were taken from distinct samples or whether the same sample was measured repeatedly
☐	☒	The statistical test(s) used AND whether they are one- or two-sided<br>*Only common tests should be described solely by name; describe more complex techniques in the Methods section.*
☐	☒	A description of all covariates tested
☐	☒	A description of any assumptions or corrections, such as tests of normality and adjustment for multiple comparisons
☐	☒	A full description of the statistical parameters including central tendency (e.g. means) or other basic estimates (e.g. regression coefficient) AND variation (e.g. standard deviation) or associated estimates of uncertainty (e.g. confidence intervals)
☐	☒	For null hypothesis testing, the test statistic (e.g. *F*, *t*, *r*) with confidence intervals, effect sizes, degrees of freedom and *P* value noted<br>*Give P values as exact values whenever suitable.*
☐	☒	For Bayesian analysis, information on the choice of priors and Markov chain Monte Carlo settings
☒	☐	For hierarchical and complex designs, identification of the appropriate level for tests and full reporting of outcomes
☐	☒	Estimates of effect sizes (e.g. Cohen's *d*, Pearson's *r*), indicating how they were calculated

Our web collection on statistics for biologists contains articles on many of the points above.

Software and code

Policy information about availability of computer code

Data collection	7T Philips Achieva scanner, 5DT Glove Ultra (Fifth Dimension Technologies), PsychToolbox-3
Data analysis	mrVista 2.0 (https://github.com/vistalab/vistasoft), Matlab version 2016b (The Mathworks), ITK-SNAP 1.6.0.1, JASP 0.12.2, afni 19.1.21, MINC 1.5.1, cbs-tools 7.4.0

For manuscripts utilizing custom algorithms or software that are central to the research but not yet described in published literature, software must be made available to editors and reviewers. We strongly encourage code deposition in a community repository (e.g. GitHub). See the Nature Research guidelines for submitting code & software for further information.

Data

Policy information about availability of data

All manuscripts must include a data availability statement. This statement should provide the following information, where applicable:

- Accession codes, unique identifiers, or web links for publicly available datasets
- A list of figures that have associated raw data
- A description of any restrictions on data availability

All data are available upon reasonable request. Source data of presented tables are provided with this paper

Field-specific reporting

Life sciences study design

All studies must disclose on these points even when the disclosure is negative.

Sample size	11 individuals participated in the study. 7 individuals participated in the haptic fMRI numerosity study. Out of these 7 individuals, 6 individuals were also scanned in the visual numerosity experiment. 5 individuals participated in a movement control experiment (3 of these participants were included in the fMRI experiments). Behavioral tests of haptic and visual numerosity perception included 5 individuals (1 of these participants was included in the fMRI experiments).<br>Our analyses aim to show significant results at the individual participant level (no averaging is needed). A few subject are then used to show that the results can be generalized, and that they are consistent in terms of the activated brain areas. Similar sample size was used in previous papers (Huth, Nature, 2016; Harvey et al., Science 2013, PNAS 2015, Human Nature Behavior 2017).
Data exclusions	After the fit of the numerosity model we excluded from further analysis data points where numerosity preference was beyond the range of the presented stimuli or when the variance explained of the model was lower than 30% or when the cross validated data had lower variance explained than a regular on-off GLM analysis. In the behavioral experiments we excluded measurements where the responses of the perceived numerosity wasn't captured in time.
Replication	The findings of haptic and visual numerosity maps are shown at the single subject level.
Randomization	In the haptic fMRI experiment and the behavioral experiment using the glove, the stimuli conditions between sessions were randomized. The behavioral experiments of numerosity perception included randomized trials.
Blinding	The investigators were not blind to group allocation. However, the identity of the participants does not have any effect on data collection. The investigators were blind to the participants identity during data analyses.

Reporting for specific materials, systems and methods

We require information from authors about some types of materials, experimental systems and methods used in many studies. Here, indicate whether each material, system or method listed is relevant to your study. If you are not sure if a list item applies to your research, read the appropriate section before selecting a response.

Materials & experimental systems

n/a	Involved in the study	
☒	☐	Antibodies
☒	☐	Eukaryotic cell lines
☒	☐	Palaeontology and archaeology
☒	☐	Animals and other organisms
☐	☒	Human research participants
☒	☐	Clinical data
☒	☐	Dual use research of concern

Methods

n/a	Involved in the study	
☒	☐	ChIP-seq
☒	☐	Flow cytometry
☐	☒	MRI-based neuroimaging

Human research participants

Policy information about studies involving human research participants

Population characteristics	Haptic numerosity study conducted in the MRI (n=7, 1 female, 1 left handed, mean age 33, age range 26-46). Movement control experiment (n=5, 2 male,s 1 left handed, mean age 32, age range 25-38). Behavioral tests of haptic and visual numerosity perception (n=5, 1 male, 2 left handed, mean age 29, age range 25-35).
Recruitment	All participants volunteered to take part in the experiments. The participants were recruited through the research facility. Results of the experiment are not expected to be biased based on sample selection or participants knowledge of the experimental aims or design.
Ethics oversight	All experimental procedures were cleared by the ethics committee of University Medical Centre Utrecht.

Note that full information on the approval of the study protocol must also be provided in the manuscript.

Magnetic resonance imaging

Experimental design

Design type	task fMRI
Design specifications	The numerosity experiments, either visual or haptic, included 4-8 repeated runs in each scanning session. Each stimulus configuration (visual, haptic) was acquired in 1 or 2 scanning sessions on different days. In general, the visual numerosity condition included 8 functional repetitions, and the haptic numerosity included 8 or 16 repetitions per

condition. The haptic numerosity runs included 208 TRs and lasted 312 seconds. The visual numerosity runs included 182 TRs and lasted for 354.9 seconds.

Behavioral performance measures: The visual numerosity experiment recorded the number of correct responses. Behavior was reported in previous studies as evidence they looked at the stimuli.

Acquisition

Imaging type(s)	functional
Field strength	7T
Sequence & imaging parameters	Functional runs: 32 channel head coil, isotropic resolution of 1.75 mm3; TR/TE= 1950/25 and TR/TE= 1500/25 for visual and haptic numerosity functional scans, respectively; flip angle=70; multiband factor=2. The haptic data included 52 slices and the visual data 64 slices.
Area of acquisition	Whole brain (almost- missing frontal pole and cerebellum).

Diffusion MRI ☐ Used ☒ Not used

Preprocessing

Preprocessing software	mrVista (https://github.com/vistalab/vistasoft), Matlab version 2016b (The Mathworks)
Normalization	Analyses were performed in the subject's native space.<br>In order to localize the average position of the haptic numerosity maps across participants we transformed the centre of the maps of each subject into MNI space using MINC toolkit (http://packages.bic.mni.mcgill.ca), applying rigid alignment and linear scaling. We then averaged the resulting MNI coordinates across participants.<br>In order to show the overlap of the maps across participants we also transformed each subject's anatomical MRI data, together with the map surfaces, centers and "end" borders to the Talairach N27 surface. This was done using AFNI's 3dAllineate and 3dNwarpApply tools.
Normalization template	Talairach N27 - though most analyses were done in the participant's native space
Noise and artifact removal	Functional runs were corrected for head movement and motion using AFNI. No temporal and spatial smoothing was applied.
Volume censoring	The first 6 timeframes of the visual numerosity functional scans scan were discarded to ensure steady-state magnetization.<br>The first 8 timeframes were discarded from the haptic numerosity functional scans.

Statistical modeling & inference

Model type and settings	population receptive field modeling
Effect(s) tested	model fits with variance explained higher than 30%

Specify type of analysis: ☒ Whole brain ☐ ROI-based ☐ Both

Statistic type for inference (See Eklund et al. 2016)	voxel-wise
Correction	The voxel-wise population receptive field model fits were not corrected for multiple comparisons, but all the following statistics on the numerosity maps were.

Models & analysis

n/a	Involved in the study
☒	☐ Functional and/or effective connectivity
☒	☐ Graph analysis
☐	☒ Multivariate modeling or predictive analysis

Multivariate modeling and predictive analysis: population receptive field modeling
